# A Novel Design Strategy for Suppressing Efficiency Roll-Off of Blue Thermally Activated Delayed Fluorescence Molecules through Donor–Acceptor Interlocking by C–C Bonds

**DOI:** 10.3390/nano9121735

**Published:** 2019-12-05

**Authors:** Tae Hui Kwon, Soon Ok Jeon, Masaki Numata, Hasup Lee, Yeon Sook Chung, Jong Soo Kim, Soo-Ghang Ihn, Myungsun Sim, Sunghan Kim, Byeong Moon Kim

**Affiliations:** 1 Department of Chemistry, Seoul National University, Seoul 08826, Korea; 2 Samsung Advanced Institute of Technology, Samsung Electronics Co., Ltd., 130 Samsung-ro, Suwon-si, Gyeonggi-do 16678, Korea; 3 Samsung R&D Institute Japan, 2-7 Sugasawa-cho, Tsurumu-ku, Yokohama 230-0027, Japan

**Keywords:** organic light-emitting diode (OLED), thermally activated delayed fluorescence (TADF), blue emitter, C–C bond, synthesis, external quantum efficiency (EQE), efficiency roll-off

## Abstract

The short material lifetime of thermally activated delayed fluorescence (TADF) technology is a major obstacle to the development of economically feasible, highly efficient, and durable devices for commercial applications. TADF devices are also hampered by insufficient operational stability. In this paper, we report the design, synthesis, and evaluation of new TADF molecules possessing a sterically twisted skeleton by interlocking donor and acceptor moieties through a C–C bond. Compared to C–N-bond TADF molecules, such as CPT2, the C–C-bond TADF molecules showed a large dihedral angle increase by more than 30 times and a singlet–triplet energy-gap decrease to less than 0.22 eV because of the steric hindrance caused by the direct C–C bond connection. With the introduction of a dibenzofuran core structure, devices comprising BMK-T317 and BMK-T318 exhibited a magnificent display performance, especially their external quantum efficiencies, which were as high as 19.9% and 18.8%, respectively. Moreover, the efficiency roll-off of BMK-T318 improved significantly (26.7%). These results indicate that stability of the material can be expected through the reduction of their singlet–triplet splitting and the precise adjustment of dihedral angles between the donor–acceptor skeletons.

## 1. Introduction

Organic light-emitting diodes (OLEDs) are considered powerful components of lighting devices. They have enormous potential applications to general lighting, larger displays, and flexible displays because of their superior color properties, thinness, and flexibility [1,2,3,4,5,6]. However, traditional OLEDs have limitations, such as low efficiency, because only 25% of the singlet excitons can be harvested in accordance with quantum mechanics [7,8,9]. Tireless efforts toward increasing the efficiency and stability of OLEDs have continued, and various photophysical phenomena in the field of OLEDs have been discovered, such as singlet fission, triplet–triplet annihilation, and thermally activated delayed fluorescence (TADF) [7]. In particular, TADF emitters allow an internal quantum efficiency theoretically up to 100% by harvesting both singlet and triplet excitons via reverse intersystem crossing (RISC). Thus, TADF technology has attracted significant attention [10,11].

To facilitate the RISC process of the TADF phenomenon, it is necessary to have a small energy gap between the lowest singlet energy and triplet energy in excited states, which is typically less than 0.37 eV [12,13,14,15,16]. The backbones of the TADF emitters are made up of electron-donating and -accepting moieties for spatial separation of highest occupied molecular orbital (HOMO) and lowest occupied molecular orbital (LUMO) distributions. Weak overlap of HOMO and LUMO tends to lead to small singlet–triplet energy gaps (Δ*E*_ST_) [17,18,19]. Because generally used donor units contain aniline- and carbazole-type electron-rich functional groups, the bridge between the donor and acceptor moiety is usually interlocked by a C–N bond [20,21,22,23,24,25,26,27,28,29]. The carbazole-type donor moiety can be readily introduced through a C–N bond connection to the main building block, typically via nucleophilic aromatic substitution reactions (S_N_Ar). The average bond dissociation energy of a C–N bond (305 KJ/mol) is lower than that of a C–C bond (347 KJ/mol); therefore, the C–N bonds connected between the donor and acceptor units are considered susceptible to the degradation of TADF emitters during the photoluminescence process. In particular, deep-blue emitters have a large excitation energy, approximately 3.0 eV [11]. To alleviate possible degradation of deep-blue TADF materials, a more stable junction between the donor and acceptor groups is deemed more desirable than the C–N bond connection.

Herein, we demonstrate that a new molecular design, employing a C–C bond interlocking donor and acceptor moieties, can lead to the stability improvement of blue TADF emitters. Our modelling studies on a series of C–C bonding TADF emitters suggested lower calculated Δ*E*_ST_ values, which was one of the most important factors for increased TADF phenomenon over those of the general C–N-bond blue TADF emitters (e.g., the Δ*E*_ST_ values of BMK-T138 and CPT2 were 0.10 and 0.32 eV, respectively) [28,29]. Therefore, we designed and synthesized four new TADF emitters having interlocked acceptor units on their carbazole or dibenzofuran backbone structures via C–C bond connections. Between the two backbone structures, those containing the dibenzofuran moiety were characterized by short delayed fluorescence lifetimes (*τ*_DF_), owing to high kRISC values. Thus, compounds equipped with the dibenzofuran core structure were expected to be even more stable than those with the carbazole structure. Newly designed TADF emitters consist of an interlocked acceptor at the central core, coupled with a carbazole or dibenzofuran donor in the backbone structure for a small Δ*E*_ST_. Restricted rotation of the central core via a C–C-bonding architecture leads to a large dihedral angle between the donor and acceptor because of the large steric hindrance, affecting the short delayed fluorescence lifetime (*τ*_DF_) [30].

## 2. Materials and Methods

### 2.1. General Information

Chemicals were purchased from Sigma-Aldrich Co. (Yongin, Korea), Tokyo Chemical Industry Co. (Seoul, Korea), and Wako Pure Chemical Industries Ltd. (Seoul, Korea), and they were used without further purification. The organic materials used in this study that did not have precedent syntheses were synthesized and characterized according to the methods described in the Supplementary Information. The ^1^H and ^13^C NMR spectra were measured using an Agilent 400-MR DD2 magnetic resonance system (400 MHz) spectrophotometer (Seoul, Korea) or a Bruker ASCEND 500 (500 MHz) spectrophotometer (Seongnam, Korea). Chemical shifts were measured as parts per million (*d* values) from tetramethylsilane as an internal standard at probe temperature in CD_2_Cl_2_, CDCl_3_, or DMSO-D_6_ for neutral compounds. Coupling constants were provided in Hz, with the following spectral pattern designations: s, singlet; d, doublet; t, triplet; q, quartet; quint, quintet; m, multiplet; br, broad; and app, apparent. The matrix-assisted laser desorption/ionization-time of flight (MALDI-TOF) mass spectra were recorded using a Bruker Ultraflex III TOF/TOF 200 spectrometer (Seongnam, Korea). The ultraviolet–visible (UV-vis) spectra were obtained by means of a Varian model UV–vis near-infrared spectrophotometer 5000 (Seoul, Korea), and the fluorescence spectra were measured on a HITACHI F7000 spectrometer (Seoul, Korea) for the solution states. The UV–vis absorption and solution photoluminescence (PL) emission spectra of materials were obtained from a dilute toluene solution (1 × 10^−5^ M), whereas the solid PL spectra were obtained from thin films prepared by vacuum evaporation. Triplet energy values of the TADF materials were obtained from the photoluminescence spectra at (Suwon, Korea) 77 K using liquid N_2_.

### 2.2. Synthesis

Synthetic details and spectral analysis data of all products are included in the electronic supplementary information (ESI).

### 2.3. Device Fabrication and Measurement

The organic layers were deposited on precleaned In_2_O_5_Sn (ITO) glass substrates through the use of a thermal evaporation system having a vacuum pressure of <1.0 × 10^−6^ torr. A 1 nm thick 8-hydroxyquinolinolatolithium (Liq.) and a 100 nm thick Al (or Ag) were deposited as a cathode via thermal evaporation. The deposition rates of the organic and metal layers were about 0.1 and 0.5 nm s^−1^, respectively. That of the Liq. layer was about 0.01 nm s^−2^. The active device area of 4 mm^2^ was defined by the overlapped area of the ITO and Al electrodes. The hole-only device structure was ITO/dipyrazino[2,3-f:2′,3′-h]quinoxaline-2,3,6,7,10,11-hexacarbonitrile (HAT-CN, 10 nm)/*N*4,*N*4′-di(naphthalen-1-yl)-*N*4,*N*4′-diphenyl-[1,1′-biphenyl]-4,4′-diamine (NPB, 50 nm)/TADF material (30 nm)/NPB (10 nm)/Al. The device structure was ITO (50 nm)/HAT-CN (10 nm)/NPB (50 nm)/1,3-di(9*H*-carbazol-9-yl)benzene (mCP, 10 nm)/Host:TADF emitter (30 nm, 15 wt%)/ dibenzo[b,d]furan-2,8-diylbis(diphenylphosphine oxide) (DBFPO, 10 nm)/DBFPO:Liq (30 nm, 50 wt%)/Liq (1 nm)/Al (200 nm). The hosts were bis[2-(diphenylphosphino)phenyl] ether oxide (DPEPO) and 3’,5-di(9*H*-carbazol-9-yl)-[1,1’-biphenyl]-3-carbonitrile (mCBP-CN), and the TADF emitters were BMK-T138, BMK-T139, BMK-T317, and BMK-T318, respectively. Current, voltage, and luminance of the OLEDs were measured with a Keithley 2400 Source-Meter (Suwon, Korea) and PR-650 spectroradiometer (Suwon, Korea). Lifespan measurement of the OLEDs was performed at a constant current mode.

## 3. Results and Discussion

### 3.1. Synthesis

We selected four compounds among candidate structures suggested from virtual simulation screening. Diphenyltriazine attached at the 2- or 4-position of the backbone structures was selected as an acceptor unit, and a carbazole moiety at the 3-position of the backbone structure was chosen as an additional donor unit. The close-in location of the acceptor and additional donor was chosen for the installment of a proper twist angle between the two units.

**Scheme 1:**
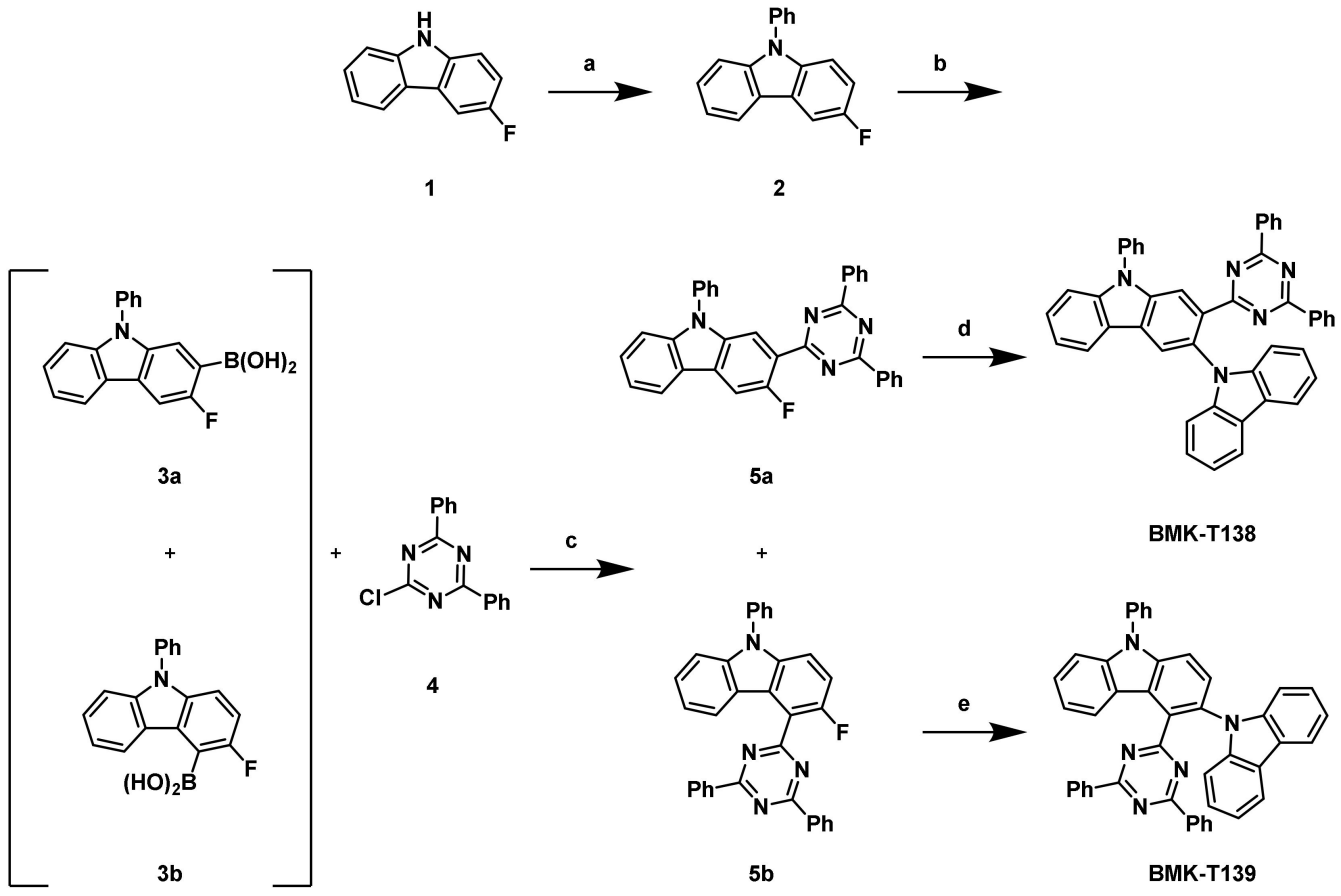
Synthetic routes of BMK-T138 and BMK-T139: (**a**) iodobenzene, K_3_PO_4_, CuI, *N*,*N*’-diacetylcyclohexane-1,2-diamine, dioxane, 80 °C; 86%; (**b**) triisopropyl borate, lithium 2,2,6,6-tetramethylpiperidide, tetrahydrofuran (THF), −60 °C, then 1 N HCl, r.t.; (**c**) Pd(OAc)_2_, tri-o-tolyphosphine, H_2_O, THF, 80 °C, 36% yield from 2 steps; (**d**) Carbazole, *t*BuOK, *N*,*N*-dimethylformamide (DMF), 160 °C, 49%; (**e**) Carbazole, *t*BuOK, DMF, 160 °C, 85%.

As shown in Scheme 1, 3-fluoro-9*H*-carbazole (1) was selected as a common starting material for the synthesis of BMK-T138 and BMK-T139. After phenylation on the carbazole nitrogen, a boronic acid unit was introduced at the carbazole ring to yield a mixture of isomers 3a and 3b in a 5:1 ratio. Suzuki–Miyaura cross-coupling reaction of the resulting boronic acid derivative with a triazine derivative (4) in the presence of a Pd catalyst provided a mixture of regioisomers, 2-(4,6-diphenyl-1,3,5-triazin-2-yl)-3-fluoro-9-phenyl-9*H*-carbazole (5a) and 3-fluoro-4-(4,6-diphenyl-1,3,5-triazin-2-yl)-9-phenyl-9*H*-carbazole (5b), which were readily separated via column chromatography. The two regioisomers, 5a and 5b, were obtained in 36% and 7% yields, respectively. Finally, S_N_Ar reactions of 5a and 5b with a carbazole unit provided the target molecules, BMK-T138 and BMK-T139, in 49% and 85% yields, respectively.

**Scheme 2:**
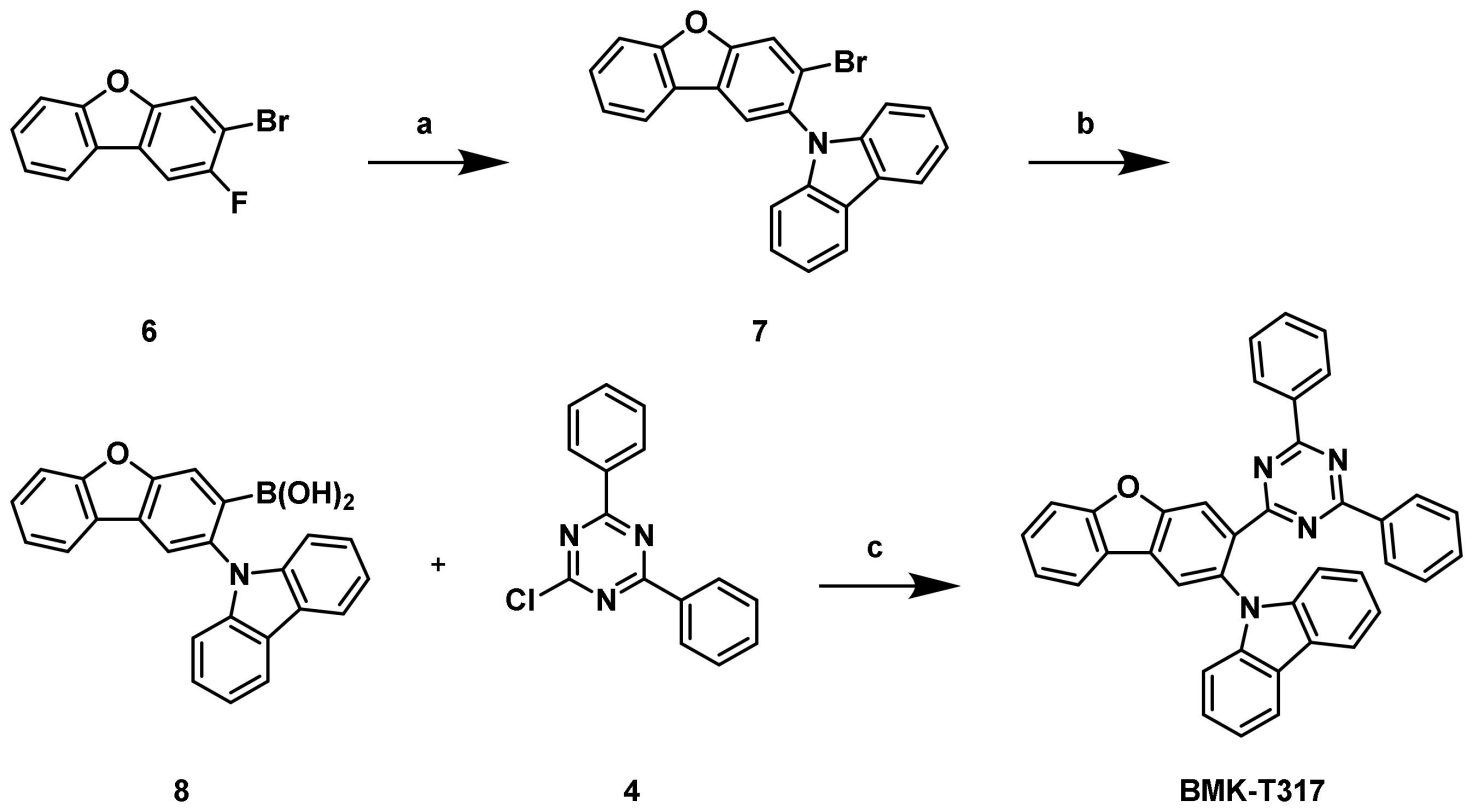
Synthetic routes of BMK-T317: (**a**) Carbazole, NaH, *N,N*-dimethyl acetamide (DMA), 160 °C, 44%; (**b**) trimethyl borate, *n*-butyllithium, THF, −78 °C, then 2 N HCl, r.t., 51%; (**c**) Pd(PPh_3_)_4_, 1 M K_2_CO_3_ in H_2_O, THF, 80 °C, 45%.

For the construction of molecules containing a dibenzofuran moiety instead of a carbazole unit at the backbone structure, a synthetic pathway featuring 3-bromo-2-fluorodibenzo[*b*,*d*]furan (6) as a key intermediate molecule was adopted, as shown in Scheme 2. Compound 6 secured the regioselective introduction of the boronic acid moiety for the construction of BMK-T317, which called for adjoining triazole and carbazole units. Compound 6 was purchased from Medigen Co. (Taipei city, Taiwan) and used without further purification. After introducing a carbazole unit at the 3-position of compound 6 via a S_N_Ar reaction, we converted the bromo group to the corresponding boronic acid (8) via borylation at the 2-bromo position, followed by hydrolysis. A Suzuki–Miyaura cross-coupling reaction was finally carried out with a triazine derivative (4), using a Pd catalyst in the same manner as shown in Scheme 1 to provide the desired compound, BMK-T317, at a 45% yield.

**Scheme 3:**
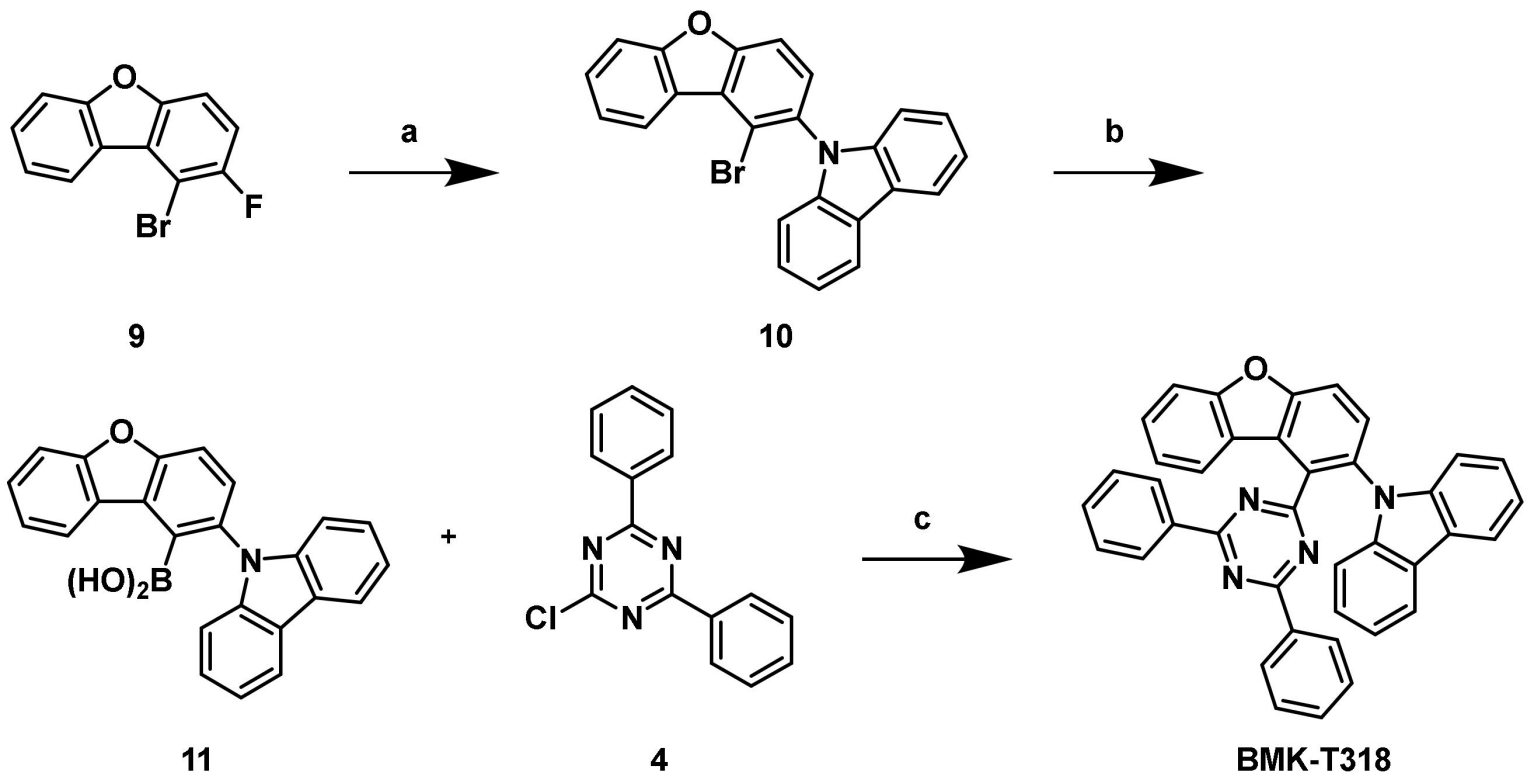
Synthetic routes of BMK-T318: (**a**) Carbazole, NaH, DMA, 160 °C, 19%; (**b**) trimethyl borate, *n*-butyllithium, THF, −78 °C, then 2 N HCl, r.t., 55%; (**c**) Pd(PPh_3_)_4_, 1 M K_2_CO_3_ in H_2_O, THF, 80 °C, 53%.

For the synthesis of BMK-T318, a new synthetic pathway was devised, which allowed the introduction of a carbazole unit before a triazine one, as shown in Scheme 3. The placement of the triazine unit in BMK-T318 required compound 9 as a key intermediate. The dibenzofuran backbone unit (9) was also purchased from Medigen Co. and used without further purification. After introduction of the carbazole unit via the S_N_Ar reaction at the 3-position of the benzofuran backbone (9), borylation was carried out at the 4-position for the final Suzuki–Miyaura cross-coupling reaction with a triazine unit (4) in the same manner as shown in Scheme 2.

All four final products were purified through flash-column chromatography followed by sublimation to ensure >99.5% purities. Detailed spectral data are included in the electronic supplementary information (ESI).

Thermal properties of the C–C bonding blue TADF emitters were characterized by thermogravimetric analysis (TGA) and differential scanning calorimetry (DSC). TGA and DSC curves of the new C–C bonding blue TADF emitters are shown in Figure S1 (ESI). Four distinct transitions were seen in the second-heating/-cooling DSC scans of BMK-T138, BMK-T139, BMK-T 317, and BMK-T318. These materials showed glass transition temperatures (*T*_g_) of 133, 130, 116, and 87.9 °C, respectively. The decomposition temperatures (*T*_d_, corresponding to 5% weight loss) of C–C bonding blue TADF emitters were in the range of 325 to 409 °C, which demonstrated their thermal robustness. The fact that apparent complete thermal decomposition with zero% weight remaining was observed in the TGA curves suggests that the materials could be readily evaporated to form thin films.

### 3.2. Calculation

**Figure 1:**
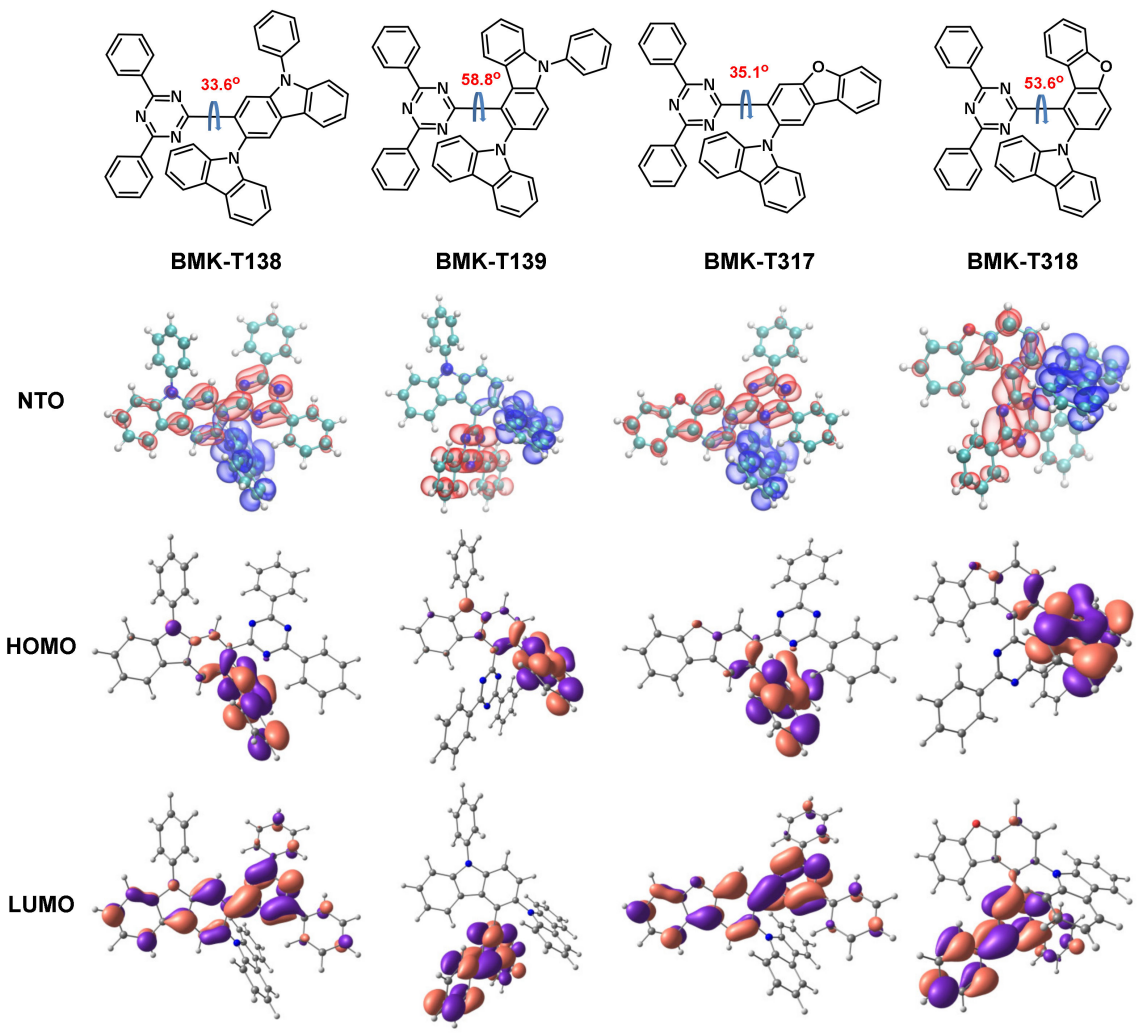
Chemical structures and dihedral angle, natural transition orbital (NTO) hole (blue) and acceptor (red), and HOMO and LUMO orbital distributions.

The molecular orbital distributions of the emitter molecules are shown in Figure 1. As can be expected from the molecular structures, the HOMOs were localized on the carbazole-donor unit, whereas the LUMOs were dispersed over the diphenyl triazine-acceptor unit. Strengths of donor and acceptor of the materials were affected by their energy levels. Especially, the dibenzofuran core structure tended to be the weak electron transport (ET) type, compared to that of the carbazole core structure. Therefore, the energy band of dibenzofuran-based C–C bond molecules was lower than that of the carbazole-based C–C bond molecules to maintain the energy band gap (Table 1). This led to a lower HOMO energy level to balance the exciton in emission layer (EML) and to maintain blue emission. Four TADF emitters interlocking through the C–C bonds were equipped with greater dihedral angles, warranting lower Δ*E*_ST_ values than those of TADF emitters interlocking through C–N bonds. In these molecules, hindered bond rotation caused by repulsive steric interaction not only partly interrupted the conjugation system, they also caused sectional deformation of both the linear and planar molecular structures, which inevitably affected the dihedral angle and emitter characteristics, such as Δ*E*_ST_ and oscillator strength (*f*). Therefore, the placement of a C–C bond on the donor–acceptor connection led to low efficiency roll-off for the newly designed material.

**Figure 2:**
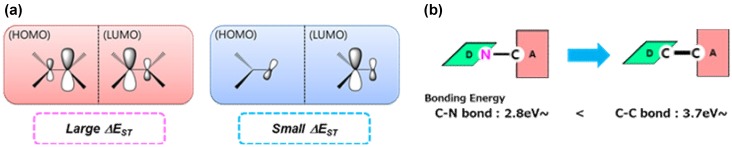
(**a**) Design principle for Δ*E*_ST_ in orbital geometry. (**b**) A diagram of bonding energy of C–N and C–C bonds.

Typical TADF emitter design requires proper placement of electron donor and acceptor moieties, which are connected in a twisted geometry (Figure 2a) to minimize the HOMO–LUMO overlap so that the emission efficiency can be maximized because of the lower S1–T1 energy gap (Δ*E*_ST_) [1,2].

All four C–C bond TADF emitters were predicted to exhibit properly separated HOMOs and LUMOs as well as lower-energy gaps (Δ*E*_ST_) than 0.10 eV, owing to the steric hindrance. It was confirmed that the Δ*E*_ST_ values of emitters having a C–C bond connection (about 0.05–0.1 eV) were three times smaller than those of emitters having C–N bond connection (0.32 eV), as reported in the case of compound CPT2 in Adachi’s study [28]. Owing to the steric hindrance in our suggested structure, the dihedral angles of BMK-T139 and BMK-T318 were calculated to be 58.8° and 53.6°, respectively, which is much larger than those of C–N bond connecting emitters (that of CPT2 was 2.0°). The Δ*E*_ST_ values were also reduced from those of the C–N bond connecting emitters. On the other hand, the oscillator strength (*f*) value decreased significantly. Thus, the PL quantum yield (PLQY) was reduced by the influence of the reduced oscillator strength (*f*). To secure a desirable PLQY, we changed the core structure from carbazole to dibenzofuran.

Our emitter design was guided by quantum chemical consideration using time-dependent density functional theory (TD-DFT) calculations, which provided estimates of the Δ*E*_ST_ for the donor–acceptor-based emitters. We performed the TD-DFT calculations on ground-state geometries using the B3LYP [31] functional and the 6-31G* basis set in gas phase. Excited-state geometries and energies were derived by TD-DFT at the MPW1B95 [32]/6-31G(d,p) level of theory. The RISC rate, $k_{RISC}$, was computed following the Fermi golden rule [33] and Marcus theory [34,35]:

$$k_{RISC}=\frac{2\pi }{\hbar }{\left|\left\langle S_{1}|H_{SO}|T_{1}\right\rangle \right|}^{2}\sqrt{\frac{1}{4\pi k_{B}T\lambda_{S}}}exp\left[-\frac{{\left(\Delta E_{ST}+\lambda_{S}\right)}^{2}}{4\lambda_{S}k_{B}T}\right],$$

where $\left|\left\langle S_{1}|H_{SO}|T_{1}\right\rangle \right|$ is the spin–orbit coupling (SOC) matrix element between the *S*_1_ and *T*_1_ states. $k_{B}$ is the Boltzmann constant, and T is temperature, which was set to 298 K. $\Delta E_{ST}$ denotes the energy difference between the *S*_1_ and *T*_1_ states, and $\lambda_{S}$ represents the reorganization energy, which can be formulated as

$$\lambda_{S}=E_{S1}\left(T_{1}\;geometry\right)-\;E_{S1}\left(S_{1}\;geometry\right).$$

All DFT and TD-DFT computations were performed using the Gaussian 09 program, and the SOC calculations were performed using the Amsterdam Density Functional (ADF). The SOC and reorganization energy values are summarized in the supporting information (Table S1).

Based on this calculation, emitters possessing the C–C bond connections were highly advantageous in terms of material stability because their bonding energies increased. The results indicate that the calculated bonding energy values of C–N and C–C were 2.8 and 3.7 eV, respectively (Figure 2b). Among the four suggested compounds, both BMK-T317 and BMK-T318, having a dibenzofuran backbone, were predicted to have much higher values of kRISC than those of BMK-T138 and BMK-T139, having a carbazole backbone (Table 1). This indicates that BMK-T317 and BMK-T318 had a delayed fluorescence lifetime (*τ*_DF_) with a high quantum yield.

### 3.3. Photophysical Properties

Optical absorption and PL spectra of the BMK-T138, BMK-T139, BMK-T 317, and BMK-T318 in dilute toluene solution (1 × 10^−4^ M) are shown in Figure S2 and S3. The key values of the photophysical properties of the four blue TADF emitters (i.e., PL emission maximum (λ_max_^em^), *E*_S_, *E*_T_, Δ*E*_ST_, full width at half-maximum (FWHM), and PLQY) are summarized in Table 2**.** The PL emission maxima (λ_max_^em^) of BMK-T138, BMK-T139, BMK-T 317, and BMK-T319 were observed at 478, 480, 469, and 466 nm, respectively. All TADF emitters exhibited narrow FWHM of 66–75 nm. To calculate the Δ*E*_ST_ of the TADF emitters, we compared the *E*_T_, which was measured at 77 K, and the *E*_S_, which was measured at room temperature, from the onset energy of the fluorescence and phosphorescence spectra. The measured HOMO-LUMO energy levels of the TADF emitters are summarized in the supporting information (Table S2).

The *E*_S_/*E*_T_/∆*E*_ST_ values of the BMK-T138 and BMK-T139 emitters were 2.87/2.82/0.05 eV and 2.87/2.75/0.12 eV, respectively, from the onset energy of the fluorescence and phosphorescence spectra. On the other hand, BMK-T317 and BMK-T318, which used dibenzofuran-based emitters, exhibited smaller ∆*E*_ST_ values, resulting from the *E*_S_ decrease and the *E*_T_ increase. As expected from the DFT calculation, the singlet–triplet energy gaps of the emitters having C–C bonds were lower than that of the CPT2 having a C–N bond (that of CPT2 was 0.32 eV) [28].

The PLQYs of BMK-T138, BMK-T139, BMK-T 317, and BMK-T318 were found to be 0.741, 0.622, 0.697, and 0.737, respectively. To improve the RISC yield while maintaining efficient PLQY of TADF emitters, we modified the core structure from a carbazole core to a dibenzofuran core. 

Using the PLQY and transient PL data, the RISC yield was calculated for each emitter. Because the *τ*_DF_ values of the dibenzofuran-based TADF emitters decreased as expected in the calculation, the RISC yields of the dibenzofuran-based TADF emitters were shown to be higher than those of the carbazole-based TADF emitters (63.09 vs. 32.10). Additionally, large distortions of the donor from the acceptor linker increased the RISC yield with a reduced *τ*_DF_ [36].

### 3.4. Device Performances

**Figure 3:**
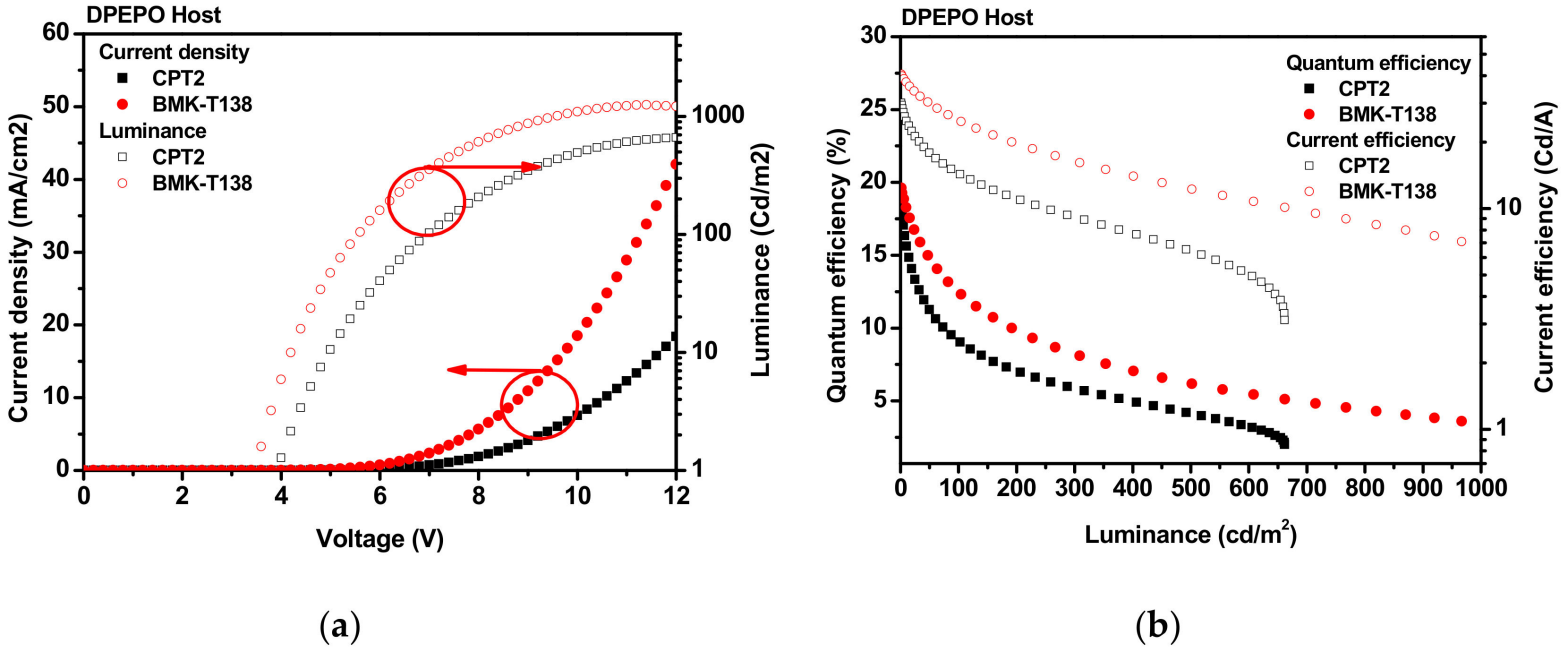
CPT2 and BMK-T138 TADF devices with DPEPO (15%) host: (**a**) current density–voltage–luminance (J–V–L); (**b**) External quantum efficiency (EQE) and current efficiency (CE) versus current density curves.

To compare the C–N and C–C bond skeleton molecules, device evaluation was conducted using a DPEPO host for CPT2 and BMK-T138 TADF emitters. Although CPT2 exhibited short delayed fluorescence lifetimes (*τ*_DF_), owing to its linear-type TADF material, it showed very efficient roll-off because of the low bond dissociation energy of the C–N bond as described in Figure 2b. Despite the long *τ*_DF_ value of BMK-T138 (163 µs), the efficiency roll-off was significantly reduced because of the large PLQY and C–C bond stability. Furthermore, the maximum external quantum efficiency of BMK-T138 was 19.6%, and the current efficiency (CE) was 40.53 cd/A, respectively. The current density–voltage–luminance (J–V–L), and quantum efficiency–luminance–current efficiency characteristics of TADF devices with C–C bond skeleton emitters are shown in Figure 3.

To summarize, the performances of the devices containing the C–C bond skeleton molecules appeared to have had the greatest effect on the stability of the material with a high PQLY (74%). Comparison of the physical properties of devices containing either a C–C bond emitter (BMK-T138) or a C–N bond emitter (CPT2) in a DPEPO host is summarized in Table 3. The advantage of the emitter equipped with a C–C bond skeleton is the increased overlap density of the HOMO/LUMO distribution with greater dihedral angle between donor and acceptor (Figure S4). By employing the dibenzofuran moiety without a phenyl linker as a core structure and increasing the overall rigidity and overlap density, we achieved short delayed fluorescence lifetimes (*τ*_DF_). Moreover, the rigid structure was expected to result in reduced nonradiative mechanisms during light emission.

Compared to the C–N bonding blue TADF emitter, those containing a C–C bond skeleton were expected to secure a high efficiency with great stability for the device. As expected, the BMK-T138 TADF emitter with a long τ_DF_ significantly improved the external quantum efficiency for the DPEPO host system. Thus, the device’s material stability was an important factor for improving device performances. Therefore, we conducted additional photophysical experiments using the mCBP-CN host instead of DPEPO [37]. Although TADF emitters with a DPEPO host are characteristically adopted for device performance tests, we examined our emitters using the mCBP-CN host, focusing on optimizing the devices with suppressed efficiency roll-off [38]. DPEPO has the advantage of high efficiency with a wide band gap and high triplet energy to boost high-efficiency TADF devices. However, it is not suitable for device stability because it is an unstable ET type host material with low stability in EML. Because DPEPO has a quite shallow LUMO for an ET-type host, all TADF emitters doped in DPEPO have deeper LUMOs than that of DPEPO. When all TADF emitters with DPEPO host have a large band gap, both hole and electron carriers are likely to be trapped inside the transport layer. As a result, these trapped carriers expose dopant molecules under electrical stress, rendering the host–dopant system less efficient.

Because mCBP-CN has an ET-type character with deep LUMO, the emission layer composed of the mCBP-CN host and TADF emitters showed better charge transport and balance than that of the DPEPO host. Therefore, we employed mCBP-CN, which is well fitted to our blue TADF emitters, having shallow LUMOs and HOMOs. Generally, the deep HOMO energy level causes a low hole injection rate in the hole-only devices (HODs). The TADF emitters having deep HOMO energy levels decreased the HOD strength. The four TADF molecules have similar tendencies, as shown in Figure S5b. Although the dibenzofuran core structure changed the energy level, it provided a highly efficient performance by minimizing the charge balance in the emission layer for TADF devices. Figure S5b shows the correlation between the hole current and HOMO energy levels.

**Figure 4:**
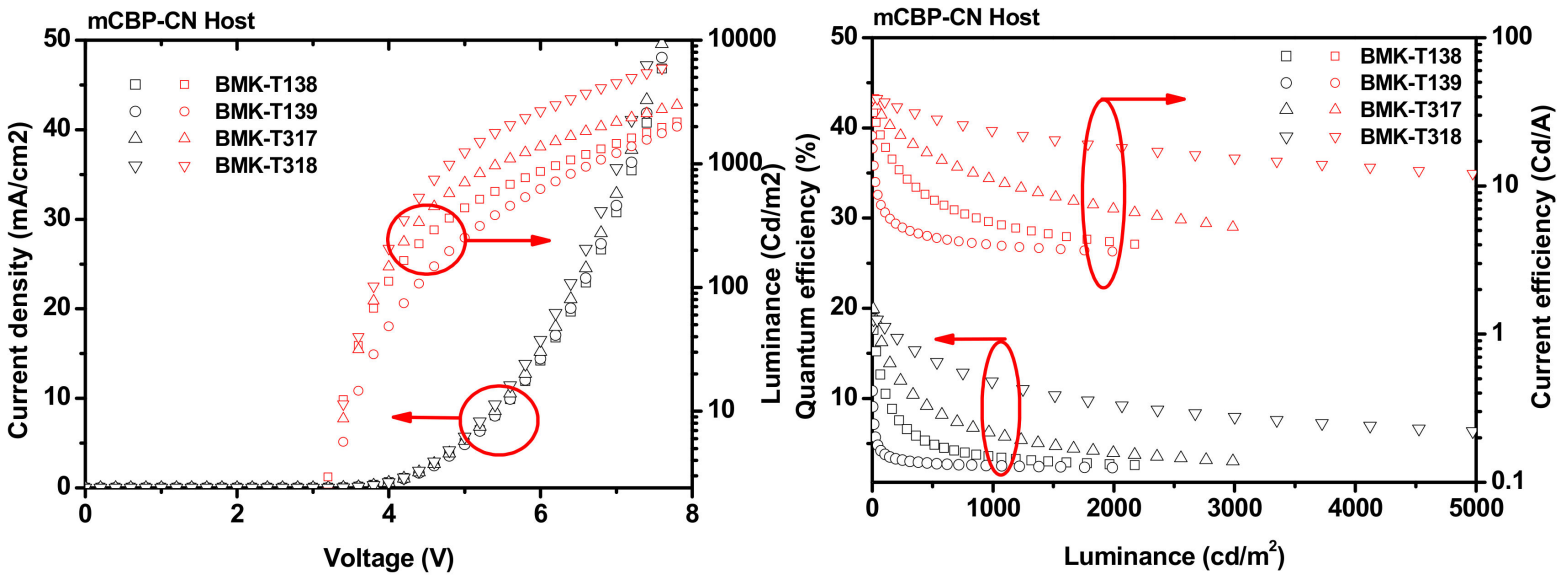
BMK-T138, BMK-T139, BMK-T317, and BMK-T318 TADF devices with an mCBP-CN (15%) host: (**a**) current (*J*–*V*–*L*); (**b**) EQE and CE versus current density curves.

TADF devices having dibenzofuran-based emitters enhanced device performance (BMK-T317 and BMK-T318) compared to those containing carbazole-based emitters, as shown in Figure 4 and Table 4. Dibenzofuran-based devices showed a significantly reduced turn-on voltage of 4.68 and 4.36 V, exhibiting increased CEs of 37.6 and 38.2 cd/A, respectively. On the other hand, carbazole-based emitters showed high turn-on voltages (5.16 and 5.71 V) and CEs of 33.8 and 21.8 cd/A, respectively. BMK-T317 and BMK-T318 also exhibited enhanced device performance, showing an EQE of 19.9% and 18.8%, respectively. In addition, the maximum luminance of BMK-T318 was much lower than that of all TADF devices, presumably owing to the low current density. BMK-T318 showed the lowest current density, and BMK-T139 showed the highest. This can be correlated with the energy levels of the core structures. The difference between the HOMO energy level of BMK-T318 and that of the mCBP-CN host was only 0.39 eV, whereas the HOMO energy level difference between BMK-T138 and mCBP-CN host was 0.50 eV. Therefore, BMK-T318 is suitable for the blue TADF because the energy barrier of BMK-T318 was the lowest.

Dibenzofuran-based materials also showed significantly enhanced efficiency roll-off because of the relatively low τ_DF_ values of BMK-T317 and BMK-T318 (51 and 21 µs, respectively), compared to the other τ_DF_ values of carbazole-based materials (BMK-T138, *τ*_DF_ = 163 µs and BMK-T139, *τ*_DF_ = 147 µs). In the case of BMK-T138, a large *τ*_DF_ value led to relatively poor efficiency roll-off than those of other C–C bond emitters. This implies that facilitating efficient host exciton transport from the recombination zone to the ground state with a high RISC yield is an important factor for securing TADF device stability. We note that RISC should be much faster than nonradioactive emission to the ground state to efficiently transfer energy from triplet to singlet. Therefore, device stability can be improved, and it can be advantageous in terms of device lifetime.

**Figure 5:**
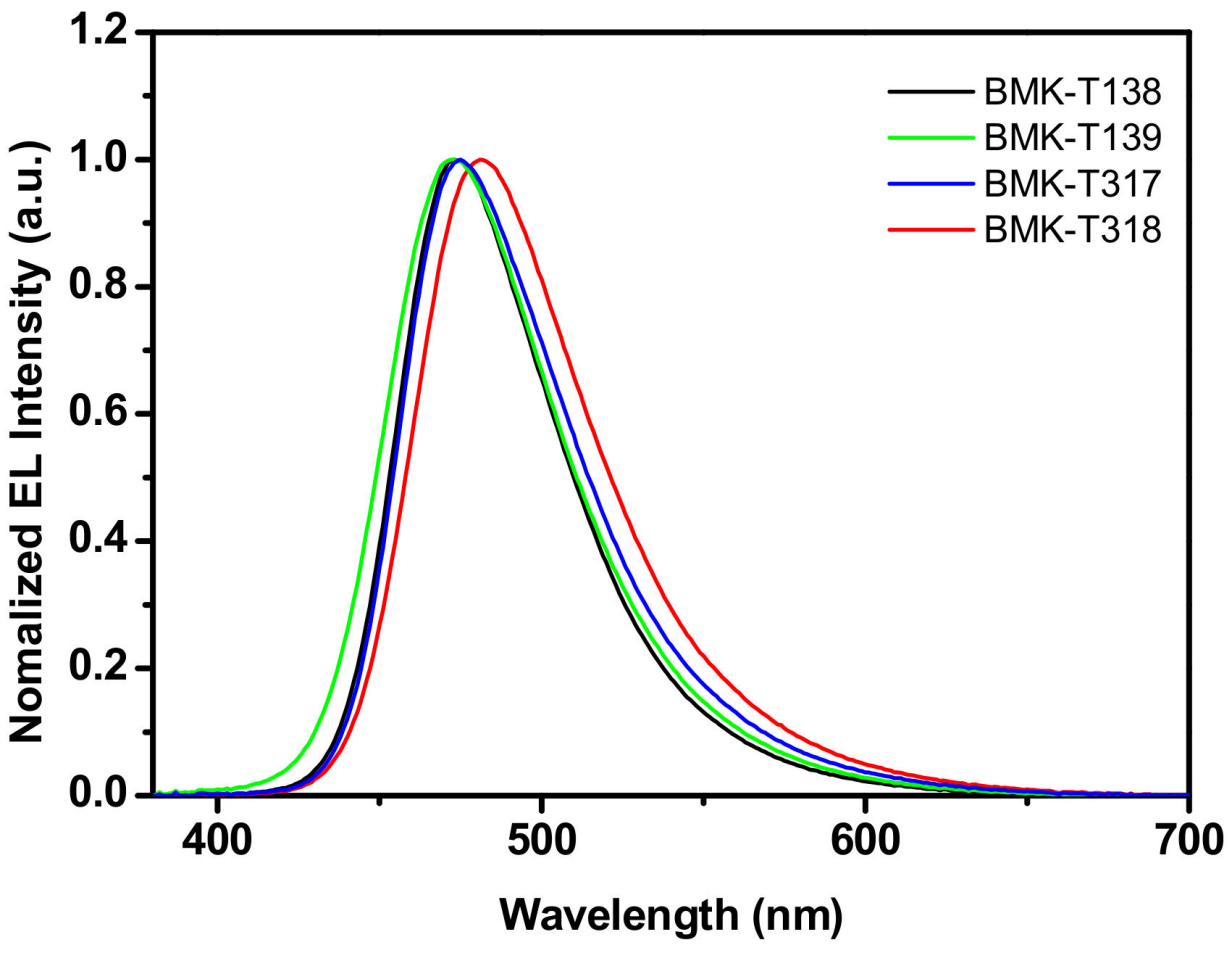
The EL spectra of BMK-T138, BMK-T139, BMK-T317, and BMK-T318 TADF devices.

Electroluminescence (EL) spectra of all devices were investigated, as shown in Table 4. The EL emission peaks were located at 473, 473, 475, and 481 nm, respectively (Table 4 and Figure 5). The EL emission peaks of compounds possessing the dibenzofuran backbone group were a bit red-shifted from the PL peak values.

## 4. Conclusions

A new design strategy of TADF emitters was proposed, entailing interlocking a donor with an acceptor through a C–C bond. The photophysical property data obtained from the synthesized compounds provided important insights on increasing stability of TADF materials during the photoluminescence process and the possibility of blue emission. A series of C–C-bond TADF emitters were introduced, featuring large dihedral angle of the central core in adjacent donor and acceptor arrangement provoking large steric hindrance. These emitters exhibited properly separated HOMO–LUMO overlap and low Δ*E*_ST_ calculation values (<0.12 eV). The photophysical properties of C–C bonding TADF emitters were characterized by a narrow FWHM of 66 nm and a blue emission of 466 nm with a short fluorescence lifetime (τ_DF_) of 21 μs in a solution state. The EL experimental results indicated a desirable EL peak of 473 nm and a high external quantum efficiency value of 19.9%. Especially clear reduction of the efficiency roll-off characteristics (26.7%) was observed in the case of dibenzofuran core structure. It is evident that the efficiency roll-off affects the operational stability of TADF emitters. Therefore, the TADF emitters having a C–C bond connection have the potential to secure a high efficiency and long life.

## Figures and Tables

**Table 1:** Density functional theory (DFT) calculation data of the thermally activated delayed fluorescence (TADF) emitters.

Name	TD-DFT ^a^							
	HOMO (eV)	LUMO (eV)	*f*	*S*1 (eV)	*T*1 (eV)	∆*E*_ST_ (eV)	*k* Reverse Intersystem Crossing (kRISC) (S^−1^)	Dihedral Angles (°)
BMK-T138	−5.16	−1.79	0.0067	2.77	2.67	0.10	8.17	33.6
BMK-T139	−5.16	−1.76	0.0043	2.86	2.81	0.05	2.99	58.8
BMK-T317	−5.30	−1.94	0.0051	2.76	2.70	0.06	5.25 × 10^5^	35.1
BMK-T318	−5.31	−1.88	0.0063	2.84	2.78	0.06	4.49 ×10^5^	53.6

^a^ The geometry in *S*_0_ was optimized by a DFT calculation.

**Table 2:** Physical properties of the TADF emitters in the solution state.

Name	Physical Properties of TADF Emitters ^a^							
	Photoluminescence (PL) (nm)	Full width at Half-Maximum (FWHM) (nm)	*E*_S_ (eV)^b^	*E*_T_ (eV)^b^	∆*E*_ST_ (eV)^c^	PL Quantum Yield (PLQY) ^d^	τ_DF_^d^ (µs)	kRISC (%)
BMK-T138	478	66	2.87	2.82	0.05	0.741	163	32.10
BMK-T139	480	72	2.87	2.75	0.12	0.622	147	50.80
BMK-T317	469	72	2.82	2.81	0.01	0.697	51	60.53
BMK-T318	466	75	2.79	2.90	−0.11	0.737	21	63.09

^a^ Measured in toluene solution. ^b^ Energy of the singlet and triplet estimated from onset of wavelength in toluene solution. ^c^ ∆*E*_ST_ = *E*_S_ – *E*_T_. ^d^ PLQY values were estimated with DPEPO host.

**Table 3:** Device performance comparison between a C–C bond emitter (BMK-T138) and a C–N bond emitter (CPT2) in the DPEPO host.

**Name**	**EQE_max_** **(%)**	**EQE ^a^** **(%)**	**CE_max_** **(Cd/A)**	**Roll-off** **(%)**	**EL** **(nm)**	**V_d_** **(V)**	**Color Coordinates**
CPT2	18.7	4.1	30.1	78.7	470	10.06	0.162, 0.206
BMK-T138	19.6	6.2	40.6	69.8	480	7.59	0.155, 0.314

^a^ EQE at luminance of 500 cd/m^2^.

**Table 4:** Device performance of the TADF emitters having an mCBP-CN host.

TADFE mitter	EQE_max_ (%)	EQE ^a^ (%)	CE_max_ (Cd/A)	Roll-off (%)	EL (nm)	*J*^b^ (mA/cm^2^)	*V*_d_^a^ (V)	Color Coordinates
BMK-T138	18.8	5.0	33.9	75.8	473	8.17	5.16	0.141, 0.240
BMK-T139	10.8	2.8	21.8	79.3	473	11.08	5.71	0.160, 0.321
BMK-T317	19.9	8.8	37.6	57.3	475	3.15	4.68	0.147, 0.228
BMK-T318	18.8	14.3	38.2	26.7	481	1.75	4.36	0.152, 0.274

^a^ EQE at luminance of 500 cd/m^2^, ^b^ Current density (*J*) at driving voltage of 5 V.

## Supplementary Materials

The following are available online at https://www.mdpi.com/2079-4991/9/12/1735/s1, Figure S1: (a) TGA and (b) DSC curves of TADF emitters, Figure S2: Optical absorption and photoluminescence spectra of (a) BMK-T138 and (b) BMK-T139, Figure S3: Optical absorption and photoluminescence spectra of (a) BMK-T317 and (b) BMK-T318, Figure S4: Overlap density-delayed fluorescence lifetimes (τ_DF_) data, Table S1: Additional theoretical computations data of the TADF emitters, Table S2: Measured HOMO–LUMO energy levels of the TADF emitters.
