# Supplementary material for: A Novel Design Strategy for Suppressing Efficiency Roll-Off of Blue Thermally Activated Delayed Fluorescence Molecules through Donor–Acceptor Interlocking by C–C Bonds
Source: Nanomaterials (Basel). 2019 Dec 5;9(12):1735. doi: 10.3390/nano9121735 (PMC6956325; doi:10.3390/nano9121735)
Supplement: Supplementary file 1 [file nanomaterials-09-01735-s001.pdf]

Supplementary information for:

A Novel Design Strategy for Suppressing Efficiency Roll-Off of Blue Thermally Activated Delayed Fluorescence Molecules through Donor–Acceptor Interlocking by C–C Bonds

Tae Hui Kwon [1,‡], Soon Ok Jeon [2,‡,*], Masaki Numata [3], Hasup Lee [2], Yeon Sook Chung [2], Jong Soo Kim [2], Soo-Ghang Ihn [2], Myungsun Sim [2], Sunghan Kim [2] and Byeong Moon Kim [1,*]

1. Department of Chemistry, Seoul National University, Seoul 08826, Korea; mkmjkm@snu.ac.kr
2. Samsung Advanced Institute of Technology, Samsung Electronics Co., Ltd., 130 Samsung-ro, Suwon-si, Gyeonggi-do 16678, Korea; hasup.lee@samsung.com (H.L.); ys2.chung@samsung.com (Y.S.C.); js100.kim@samsung.com (J.S.K.); sg.ihn@samsung.com (S.-G.I.); ms84.sim@samsung.com (M.S.); shan0819.kim@samsung.com (S.K.)
3. Samsung R&D Institute Japan, 2-7 Sugasawa-cho, Tsurumu-ku, Yokohama 230-0027, Japan; mnumata@samsung.com

‡ These authors contributed equally to this work.

* Correspondence: so.jeon@samsung.com (S.O.J.); kimbm@snu.ac.kr (B.M.K.)

Table of Contents

page

1. Experimental Sections..S3

2. NMR spectra..S6

3. Supplementary figures and tables

- **Figure S1**. TGA and DSC curves of TADF emitters S11

- **Figure S2**. Optical absorption and photoluminescence spectra of (a) **BMK-T138** and (b) **BMK-T139**.. S11

- **Figure S3**. Optical absorption and photoluminescence spectra of (a) **BMK-T317** and (b) **BMK-T318**.. S12

- **Figure S4**. Overlap density-delayed fluorescene lifetimes (τ_{DF}) data S13

- **Figure S5**. (a) Chemical structures and energy diagram of TADF devices (b) hole current versus HOMO energy S14

- **Table S1**. Additional theoretical computations data of the TADF emitters S15

- **Table S2**. Measured HOMO-LUMO energy levels of the TADF emitters.......................... S15

Experimental Sections

[Scheme 1.]

3-Fluoro-9-phenyl-9*H*-carbazole (2) : To a solution of carbazole **1** (4.63 g, 25.0 mmol) in dioxane (125 mL) was added K_3PO_4 (21.23 g, 100.0 mmol), CuI (0.24 g, 1.25 mmol), *N*,*N'*-diaminocyclohexane (0.71 g, 6.25 mmol) and iodobenzene (3.35 mL, 30.0 mmol) at r.t.. After stirring for 1 day at 90 ^oC, the resulting mixture was cooled and extracted with methylene chloride. The organic layers were dried over anhydrous MgSO4 and concentrated in vacuo. The resulting residue was purified by using flash column chromatography on silica-gel (EA:Hex=1:4) to afford carbazole **2** (5.1 g, 78%), which was used for the next step without NMR analysis data.

(3-Fluoro-9-phenyl-9*H*-carbazol-2-yl)boronic acid (3a) and **(3-Fluoro-9-phenyl-9*H*-carbazol-4-yl)boronic acid (3b) :** To a solution of carbazole **2** (10.45 g, 40.0 mmol) and tri(isopropyl)borate (11.28 g, 60.0 mmol) in anhydrous THF (200 mL) was slowly added a solution of LTMP (8.48 g, 60.0 mmol) in anhydrous THF (60 mL) at -78^oC. After stirring for 2 hours at -60 ^oC, the reaction mixture was stirred for 12 hours at room temperature. A solution of HCl (1 M in water, 100 mL) was added and the biphasic mixture was vigorously stirred for 2 hours and then extracted with ethyl acetate. The organic layers were dried over anhydrous $MgSO_4$ and concentrated in *vacuo* to afford the two regio-isomers mixture **3a** and **3b**, which were used for the next step without further purification.

2-(4,6-Diphenyl-1,3,5-triazin-2-yl)-3-fluoro-9-phenyl-9*H*-carbazole (5a) and **4-(4,6-Diphenyl-1,3,5-triazin-2-yl)-3-fluoro-9-phenyl-9*H*-carbazole (5b) :** To a solution of the above regio-isomers mixture **3a** and **3b** in THF (320 mL) was added a triazine **4** (8.58 g, 32.1 mmol), $Pd(OAc)_2$ (216 mg, 0.96 mmol), tri-o-tolyphosphine (584 mg, 1.92 mmol) and a solution of K_2CO_3 (1 M in water, 48 mL) at room temperature. The reaction mixture was allowed to warm to refulx for 6 hours. The resulting mixture was cooled and extracted with methylene chloride. The organic layers were dried over anhydrous $MgSO_4$ and concentrated in *vacuo*. The resulting residue was purified by using re-crystallization to afford carbazole **5a** (7.09 g, 36%, 2 steps) and carbazole **5b** (1.38 g, 7%, 2 steps), which were used for the next step without NMR analysis data.

2-(4,6-Diphenyl-1,3,5-triazin-2-yl)-9-phenyl-9*H*-3,9'-bicarbazole (BMK-T138) : To a solution of carbazole **5a** (2.98 g, 6.05 mmol) in DMF (18 mL) was added carbazole (6.07 g, 36.3 mmol) and tert-BuOK (3.4 g, 30.3 mmol) at r.t.. The reaction mixture was stirred at 170^oC for 18 hours. The resulting mixture was cooled and filtered using MeOH. The filter cake was dried in reduced pressure at 60 ^oC for 4 hours. The resulting residue was purified by using flash column chromatography on silica-gel (Hex:CH_2Cl_2=1:1) to afford **BMK-T138** (13.65 g, 49%).

^{1}H-NMR (400 MHz, $CDCl_3$, ppm) δ 8.06 (d, *J* = 7.6 Hz, 1H), 8.05 (d, *J* = 8.0 Hz, 2H), 7.94 (d, *J* = 7.2 Hz, 4H), 7.68-7.76 (m, 4H), 7.52-7.57 (m, 4H), 7.42 (t, *J* = 7.2 Hz, 2H), 7.28-7.38 (m, 6H), 7.19-7.26 (m, 5H). ^{13}C-NMR (100 MHz, $CDCl_3$, ppm) δ 172.7, 171.1, 142.6, 142.5, 140.3, 137.2, 135.6, 133.8, 132.1, 130.1, 128.8, 128.7, 128.2, 128.1, 127.6, 127.2, 126.3, 125.8, 123.2, 122.6, 122.5, 121.2, 120.8, 120.2, 119.2, 113.9, 110.4, 109.7. MALDI-MS Calcd: 639.24, Found: 639.26.

4-(4,6-Diphenyl-1,3,5-triazin-2-yl)-9-phenyl-9*H*-3,9'-bicarbazole (BMK-T139) : The following the same procedure as that used for the synthesis of **BMK-T138**, the reaction of carbazole **5b** (1.38 g, 2.80 mmol), carbazole (2.81 g, 16.8 mmol) and tert-BuOK (1.57 g, 14.0 mmol) in DMF gave the **BMK-T139** (1.52 g, 85%).

^{1}H-NMR (400 MHz, $CDCl_3$, ppm) δ 8.06 (d, *J* = 7.2 Hz, 4H), 7.88 (d, *J* = 7.6 Hz, 2H), 7.64-7.73 (m, 7H), 7.55-7.59 (m, 1H), 7.40-7.48 (m, 4H), 7.26-7.34 (m, 8H), 7.12-7.15 (m, 2H), 6.99-7.04 (m, 1H). ^{13}C-NMR (100 MHz, $CDCl_3$, ppm) δ 172.8, 171.3, 142.6, 142.3, 141.4, 137.1, 135.4, 132.3, 132.2, 130.2, 128.9, 128.6, 128.4, 128.3, 127.8, 127.3, 126.9, 125.5, 123.2, 122.9, 122.0, 121.9, 120.3, 120.0, 119.3, 112.3, 110.2, 110.0. MALDI-MS Calcd: 639.24, Found: 639.26.

[Scheme 2.]

9-(3-Bromodibenzo[*b,d*]furan-2-yl)-9*H*-carbazole (7) : To a solution of carbazole (3.34 g, 20.0 mmol) in DMA (20 mL) was added 60% NaH (0.80 g, 20.0 mmol) at r.t.. After stirring for 30 min, a solution of dibenzofuran **6** (2.65 g, 10.0 mmol) in DMA (10mL) was added. The reaction mixture was stirred at 160°C for 1 day. The resulting mixture was cooled and extracted with ethyl acetate. The organic layers were dried over anhydrous $MgSO_4$ and concentrated in *vacuo*. The resulting residue was purified by using flash column chromatography on silica-gel (EA:Hex=1:10) to afford carbazole **7** (1.81 g, 44%), which was used for the next step without NMR analysis data.

(2-(9H-Carbazol-9-yl)dibenzo[*b,d*]furan-3-yl)boronic acid (8) : To a solution of dibenzofuran **7** (3.70 g, 8.97 mmol) in anhydrous THF (40 mL) was slowly added 2.5 M solution of n-BuLi (7.90 mL, 19.74 mmol) at -78°C. After stirring for 1 hour, a solution of trimethyl borate (3.00 mL, 26.92 mmol) in anhydrous THF (4 mL) was added at same temperature. The reaction mixture was stirred for 1 hour and for 1 hour at room temperature. A solution of HCl (1 M in water, 20 mL) was added and the biphasic mixture was vigorously stirred for another hour and then extracted with ethyl acetate. The organic layers were dried over anhydrous $MgSO_4$ and concentrated in *vacuo*. The resulting residue was purified by using short column chromatography on silica-gel (EA:Hex=1:4) to afford boronic acid **8** (1.74 g, 51%), which was used for the next step without NMR analysis data.

9-(3-(4,6-Diphenyl-1,3,5-triazin-2-yl)dibenzo[*b,d*]furan-2-yl)-9*H*-carbazole (BMK-T317) : To a solution of boronic acid **8** (817 mg, 2.17 mmol) in THF (10 ml) was added a triazine **4** (870 mg, 3.25 mmol), $Pd(PPh_3)_4$ (250 mg, 10 mol%) and a solution of K_2CO_3 (1 M in water, 3.2 mL) at room temperature. The reaction mixture was stirred at 80°C for 12 hours. The resulting mixture was cooled and extracted with ethyl acetate. The organic layers were dried over anhydrous $MgSO_4$ and concentrated in *vacuo*. The resulting residue was purified by using flash column chromatography on silica-gel (EA:Hex=1:10) to afford **BMK-T317** (550 mg, 45%)

^{1}H-NMR (400 MHz, $CDCl_3$, ppm) δ 8.78 (s, 1H), 8.22 (s, 1H), 8.05 (d, *J* = 7.2 Hz, 2H), 7.98-8.01 (m, 5H), 7.72 (d, *J* = 8.4 Hz, 1H), 7.59 (t, *J* = 7.6 Hz, 1H), 7.40-7.48 (m, 3H), 7.29-7.34 (m, 6H), 7.20-7.24 (m, 4H). ^{13}C-NMR (100 MHz, $CDCl_3$, ppm) δ 171.9, 171.3, 157.8, 155.6, 142.3, 135.4, 135.1, 132.3, 131.8, 128.9, 128.8, 128.3, 128.0, 126.0,123.5, 123.4, 123.3, 123.0, 121.5, 120.3, 119.4, 115.8, 112.2, 109.6. MALDI-MS Calcd: 564.20, Found: 565.20.

[Scheme 3.]

9-(1-Bromodibenzo[*b,d*]furan-2-yl)-9*H*-carbazole (10) : The following the same procedure as that used for the synthesis of **7**, the reaction of carbazole (3.34 g, 20.0 mmol), 60% NaH (0.80 g, 20.0 mmol) and dibenzofuran **9** (2.65 g, 10.0 mmol) in DMA gave the dibenzofuran **10** (0.79 g, 19%) after purification by flash colmn chromatography on silica-gel (EA:Hex=1:10), which was used for the next step without NMR analysis data.

(2-(9*H*-carbazol-9-yl)dibenzo[*b,d*]furan-1-yl)boronic acid (11) : The following the same procedure as that used for the synthesis of **8**, the reaction of 2.5 M solution of n-BuLi (5.34 mL, 13.34 mmol), trimethyl borate (2.03 mL, 18.19 mmol) and dibenzofuran **10** (2.50 g, 6.06 mmol) in anhydrous THF gave the boronic acid **11** (1.25 g, 55%), which was used for the next step without NMR analysis data.

9-(1-(4,6-diphenyl-1s7,3,5-triazin-2-yl)dibenzo[*b,d*]furan-2-yl)-9*H*-carbazole (BMK-T318) : The following the same procedure as that used for the synthesis of **BMK-T317**, the reaction of boronic acid **11** (1.25 g, 3.31 mmol), triazine **4** (1.06 g, 3.98 mmol), $Pd(PPh_3)_4$ (382 mg, 10 mol%) and a solution of K_2CO_3 (1 M in water, 4.0 mL) in THF gave the **BMK-T318** (990 mg, 53%).

^{1}H-NMR (500 MHz, CD_2Cl_2, ppm) δ 8.02-0.03 (m, 5H), 7.92 (d, *J* = 6.2 Hz, 2H), 7.80 (d, *J* = 6.8 Hz, 1H), 7.78 (d, *J* = 6.3 Hz, 1H), 7.72 (d, *J* = 6.7 Hz, 1H), 7.49-7.55 (m, 3H), 7.30-7.36 (m, 6H), 7.25 (d, *J* = 6.5 Hz, 2H), 7.13-7.19 (m, 3H). ^{13}C-NMR (100 MHz, $CDCl_3$, ppm) δ 171.7, 171.4, 157.4, 156.1, 142.3, 135.2, 132.5, 132.2, 131.6, 128.9, 128.5, 128.4, 128.3, 125.8, 124.4, 123.8, 123.1, 123.0, 122.9, 120.2, 119.5, 114.5, 111.9, 109.9. MALDI-MS Calcd: 564.20, Found: 565.20.

Characterization data of products

2-(4,6-Diphenyl-1,3,5-triazin-2-yl)-9-phenyl-9*H*-3,9'-bicarbazole (BMK-T138)

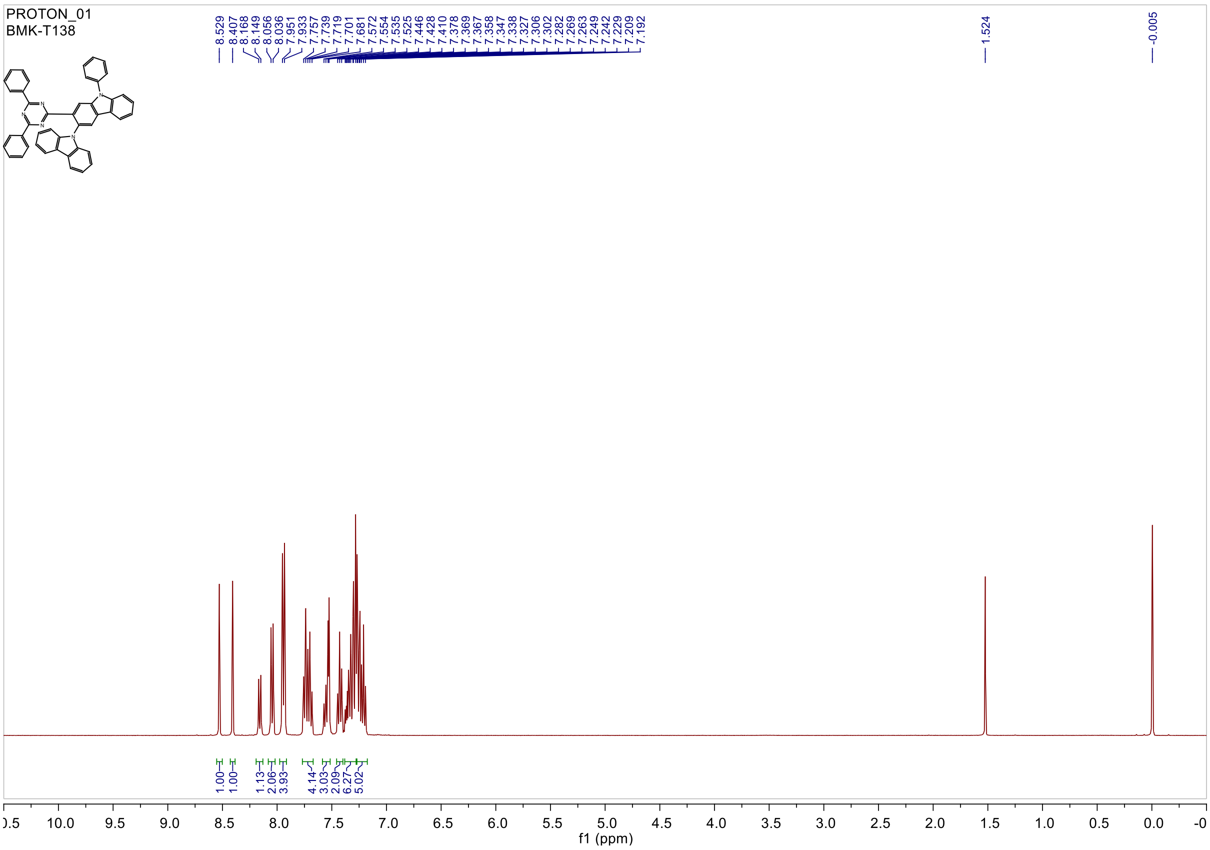

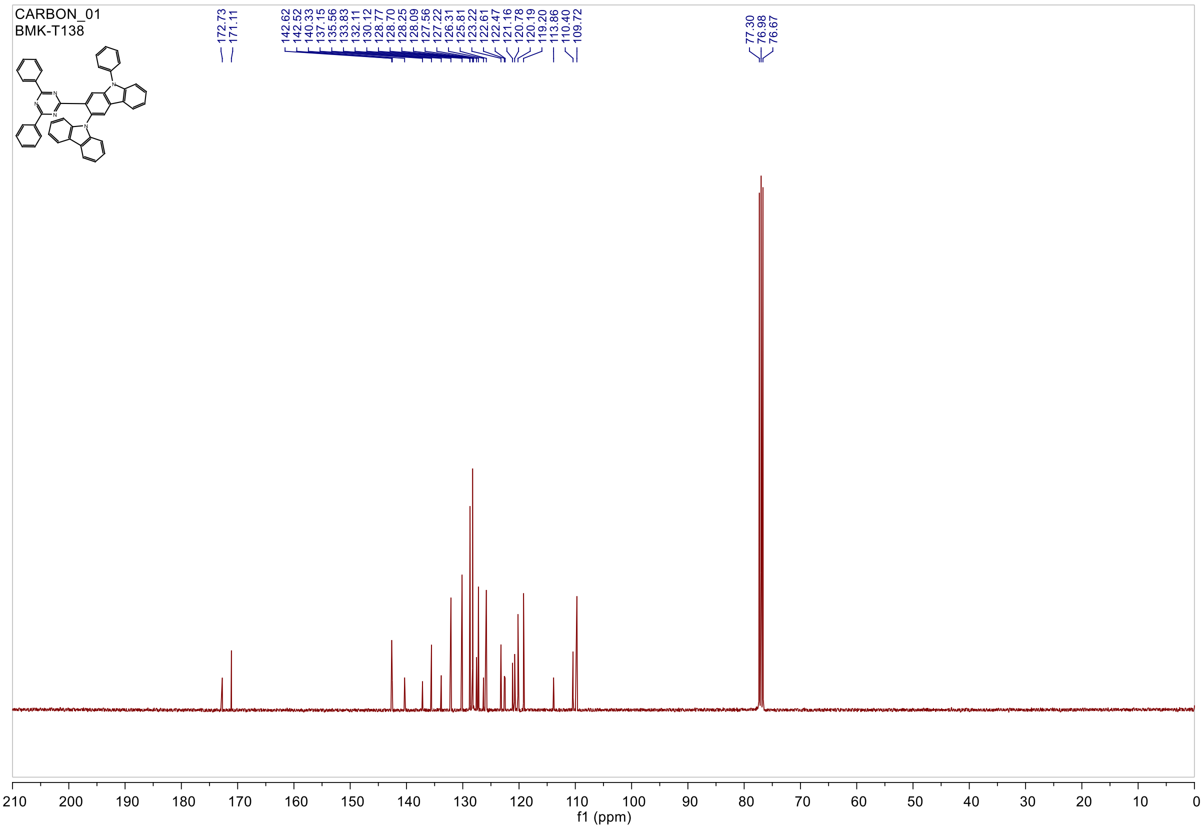

4-(4,6-Diphenyl-1,3,5-triazin-2-yl)-9-phenyl-9*H*-3,9'-bicarbazole (BMK-T139)

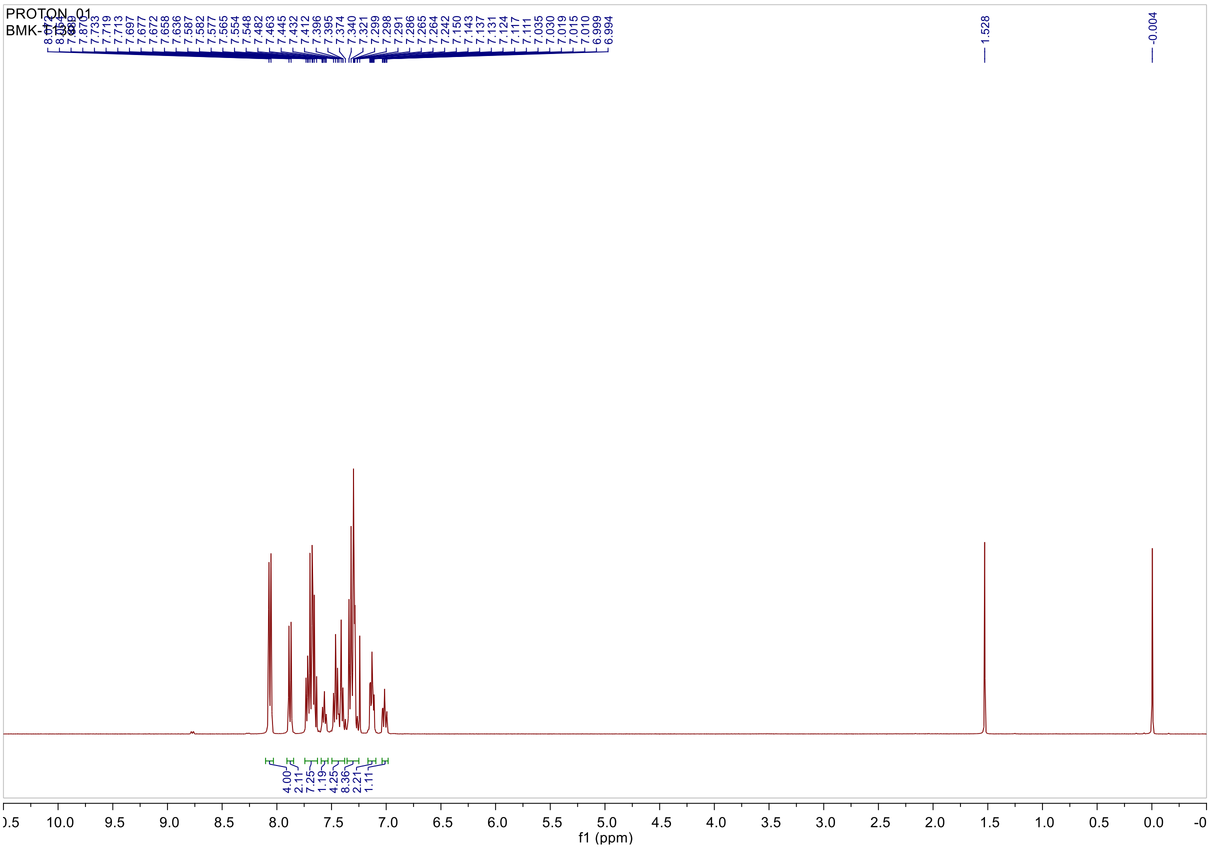

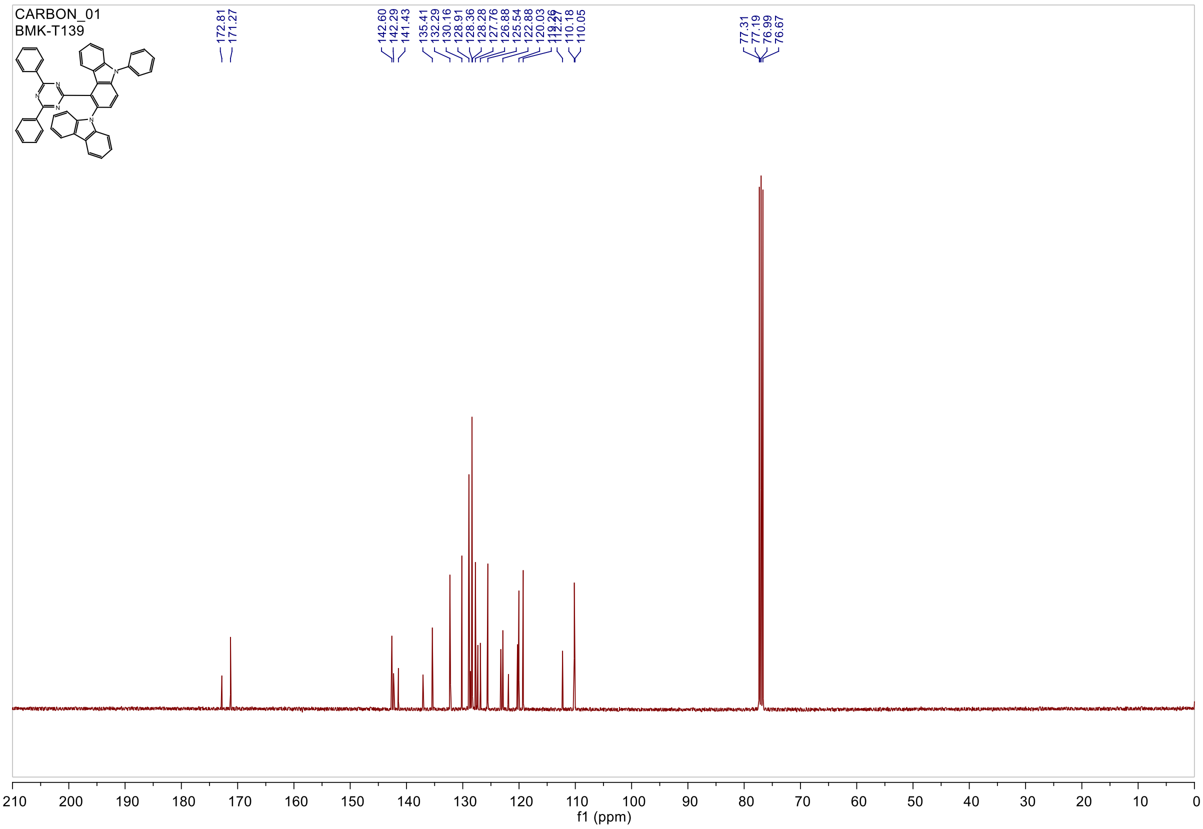

9-(3-(4,6-Diphenyl-1,3,5-triazin-2-yl)dibenzo[*b*,*d*]furan-2-yl)-9*H*-carbazole (BMK-T317)

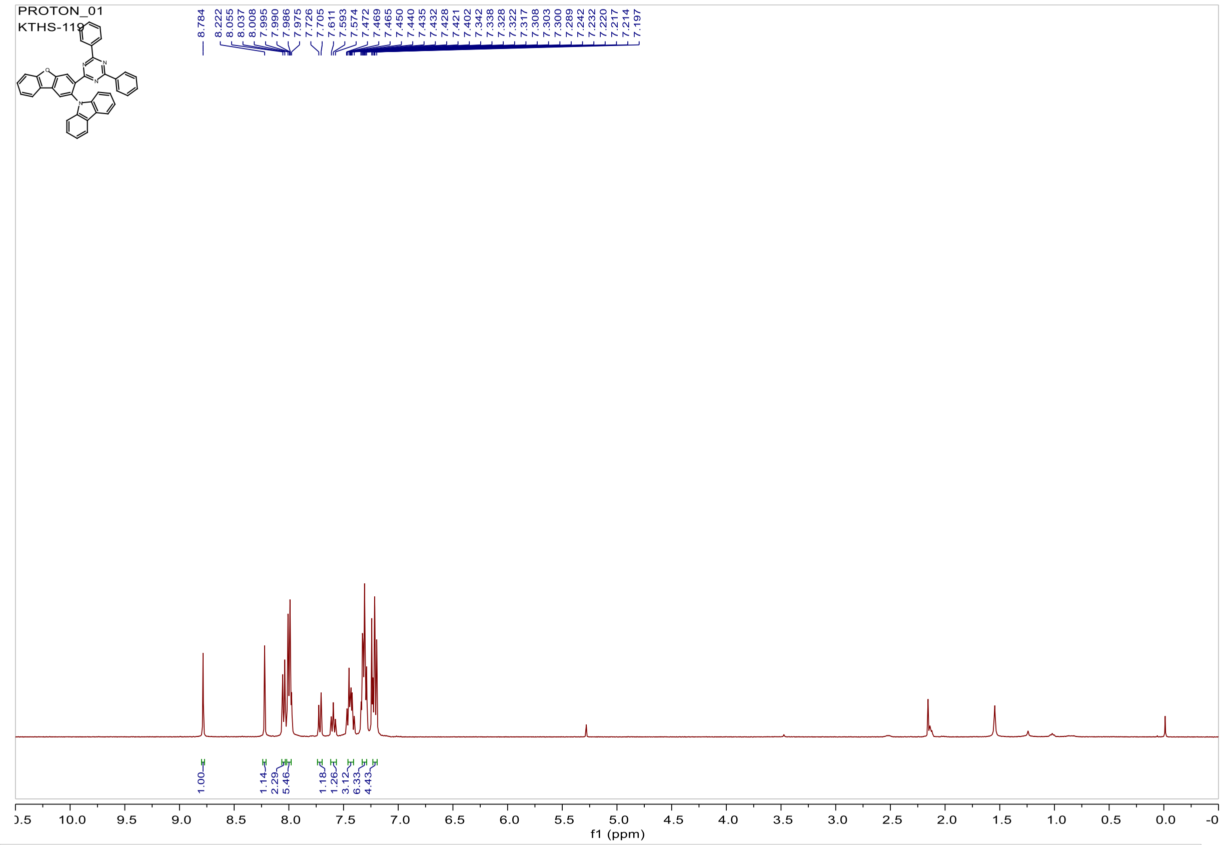

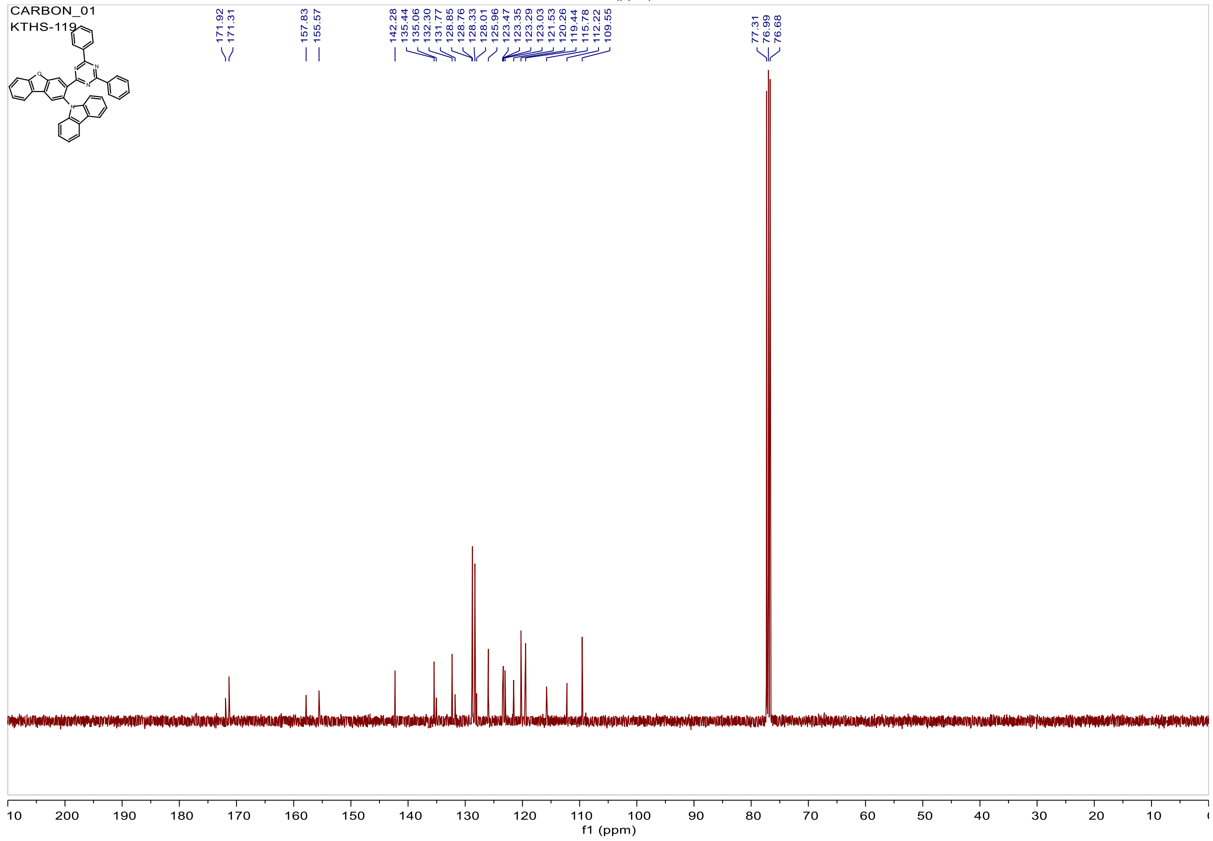

9-(1-(4,6-diphenyl-1s7,3,5-triazin-2-yl)dibenzo[*b*,*d*]furan-2-yl)-9*H*-carbazole (BMK-T318)

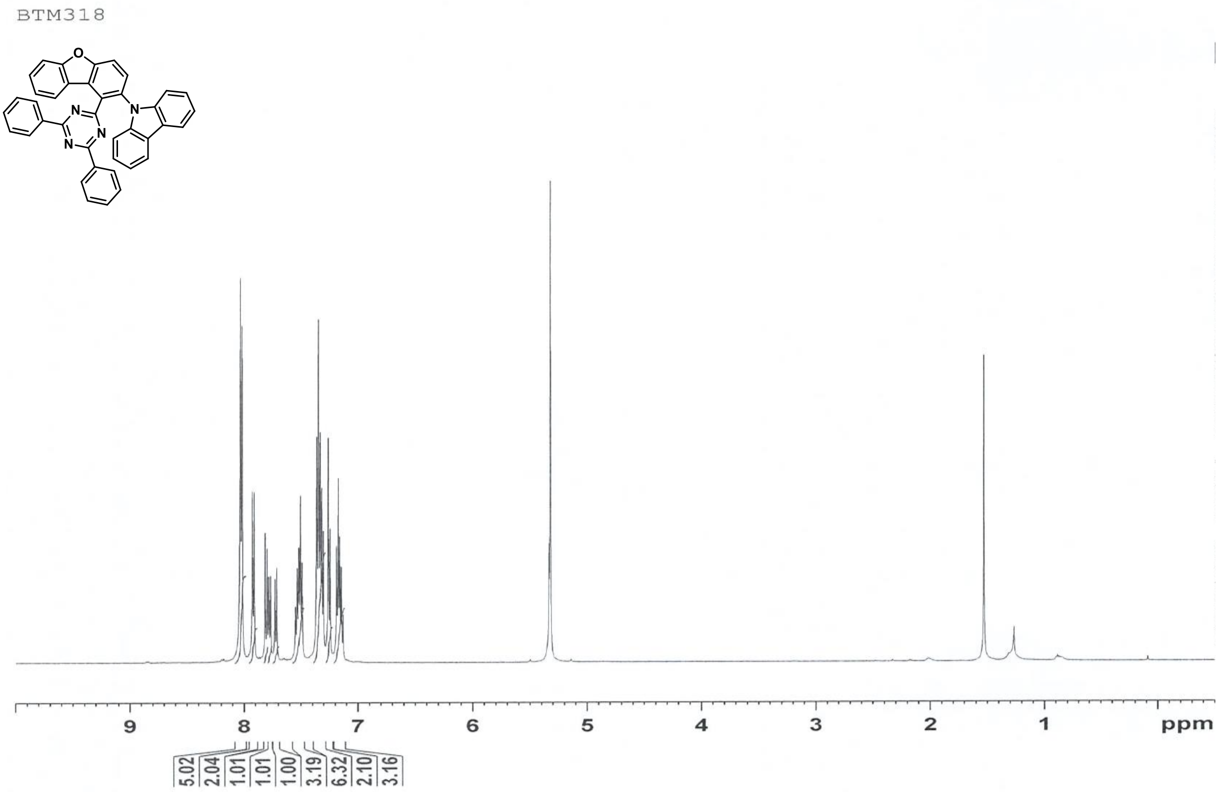
BTM318
N
N
N
O
N
9
8
7
6
5
4
3
2
1
ppm
5.02
2.04
1.01
1.01
1.00
3.19
6.32
2.10
3.16

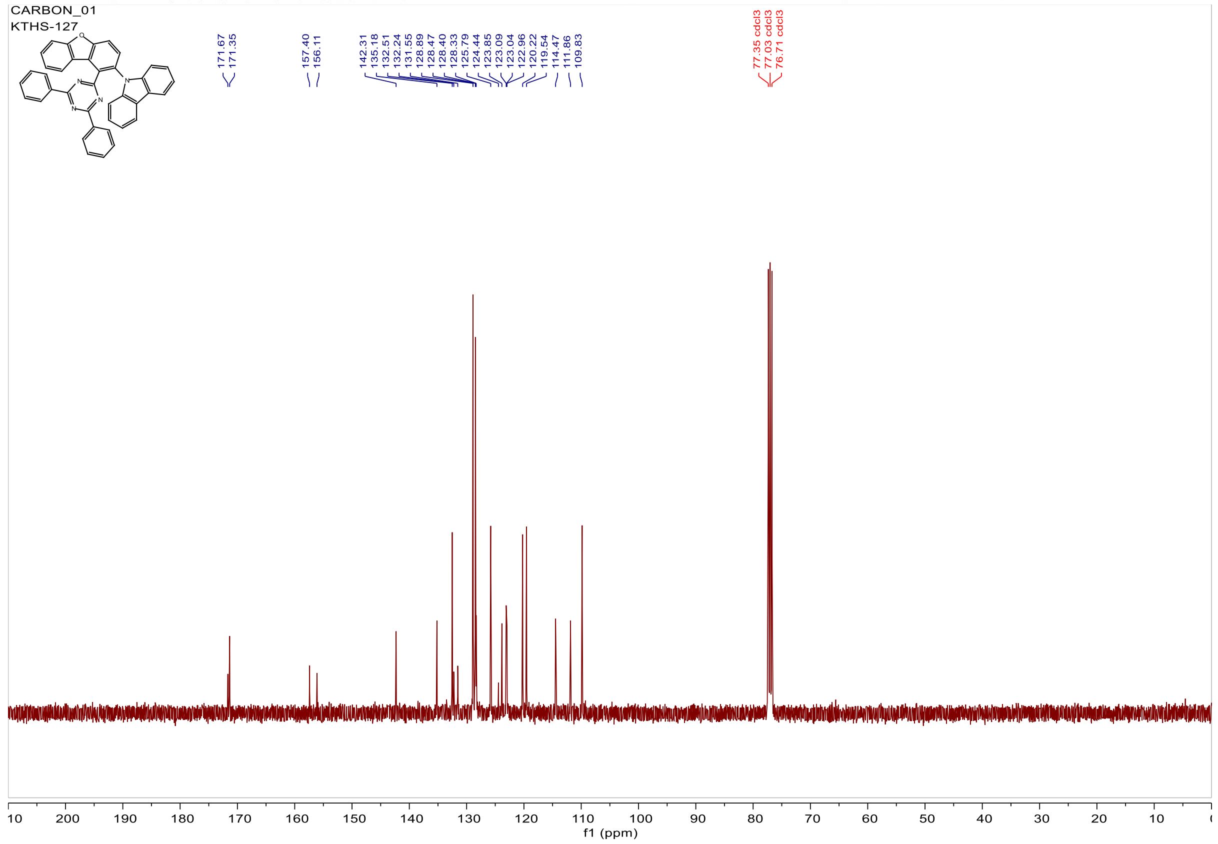
CARBON_01
KTHS-127
171.67
171.35
157.40
156.11
142.31
135.18
132.51
132.24
131.55
128.89
128.47
128.40
128.33
125.79
124.44
123.85
123.09
123.04
122.96
120.22
119.54
114.47
111.86
109.83
77.35 cdcl3
77.03 cdcl3
76.71 cdcl3
200
190
180
170
160
150
140
130
120
110
100
90
80
70
60
50
40
30
20
10
f1 (ppm)

(a)

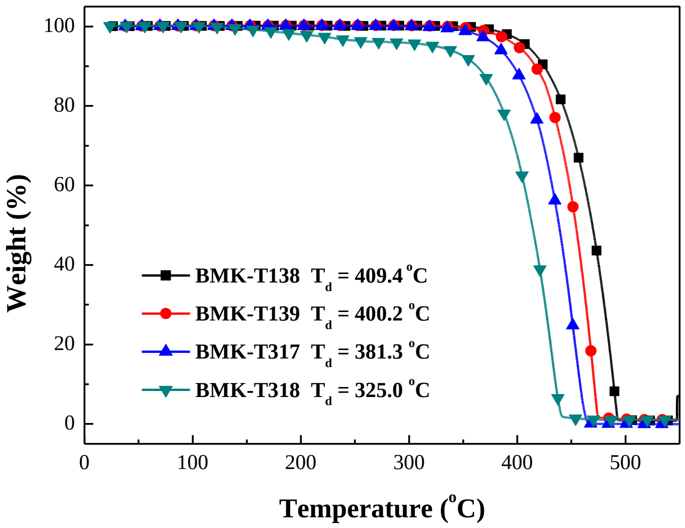

(b))

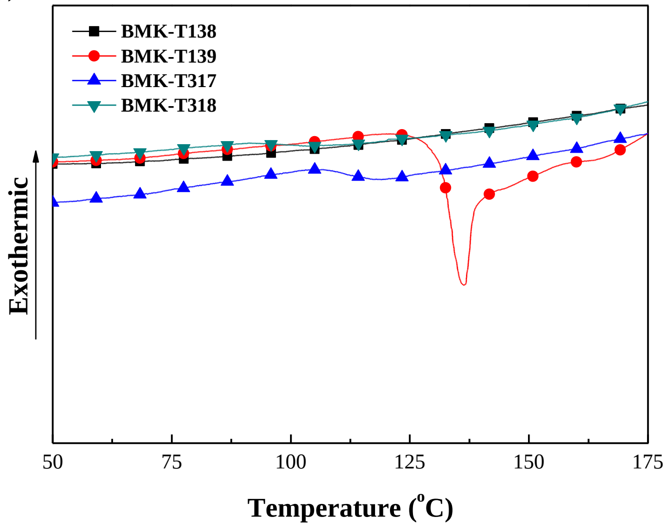

Figure S1. (a) TGA and (b) DSC curves of TADF emitters.

(a)

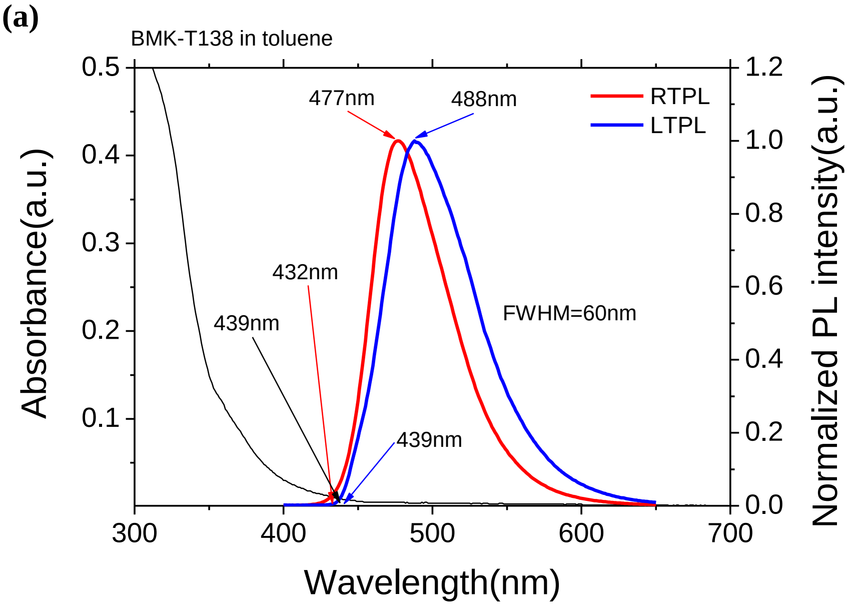

(b))

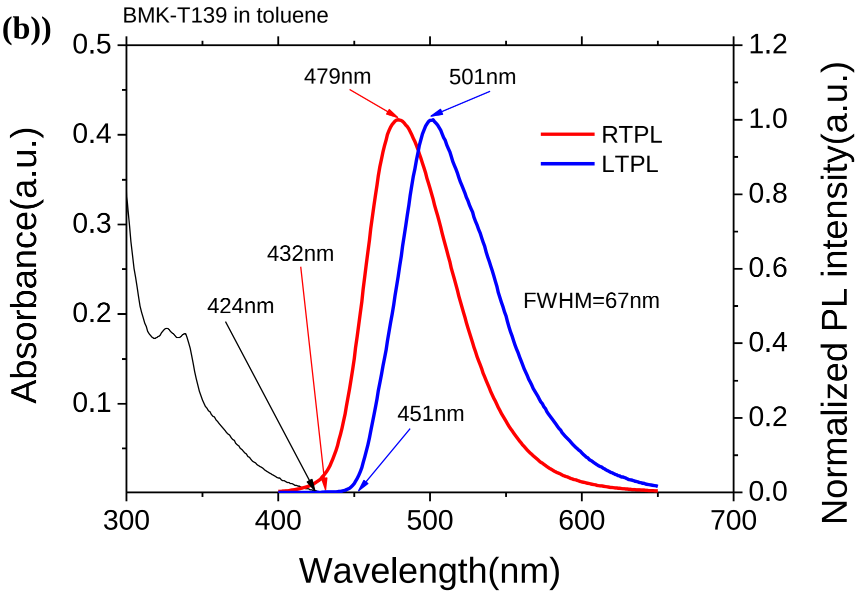

Figure S2. Optical absorption and photoluminescence spectra of (a) **BMK-T138** and (b) **BMK-T139.**

(a)

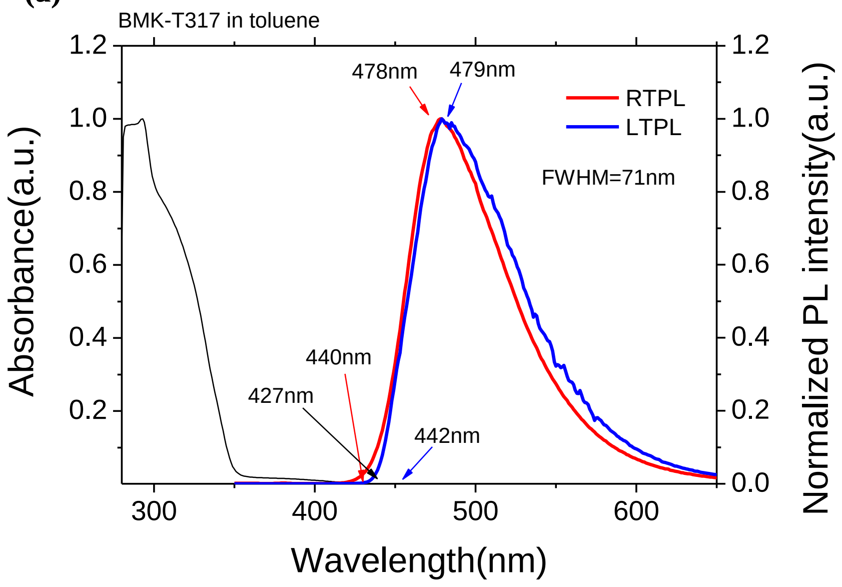

(b))

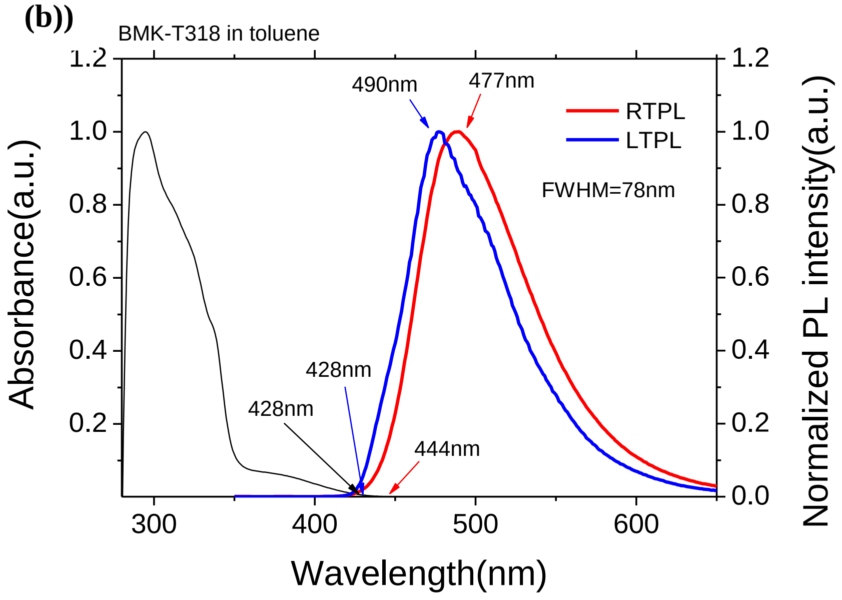

Figure S3. Optical absorption and photoluminescence spectra of (a) **BMK-T317** and (b) **BMK-T318**.

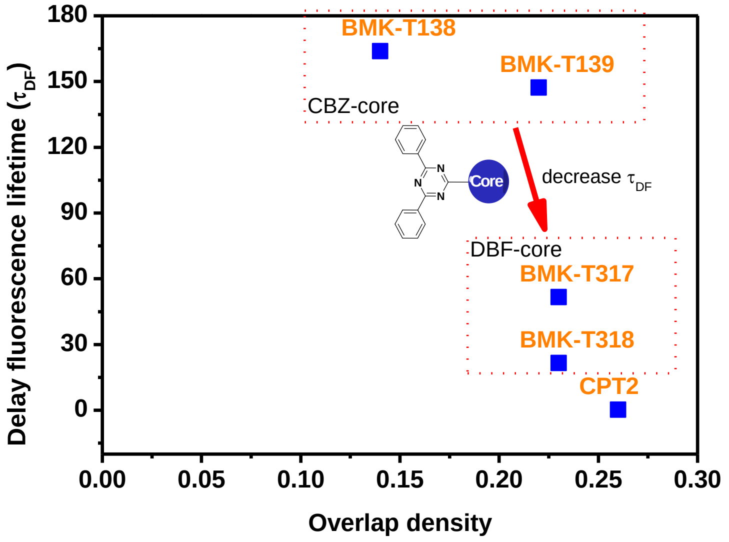

Figure S4. Overlap density-delayed fluorescence lifetimes (τ_{DF}) data

The τ_{DF} values of the dibenzofuran-based TADF emitters were lower than those of carbazol-based TADF emitters.

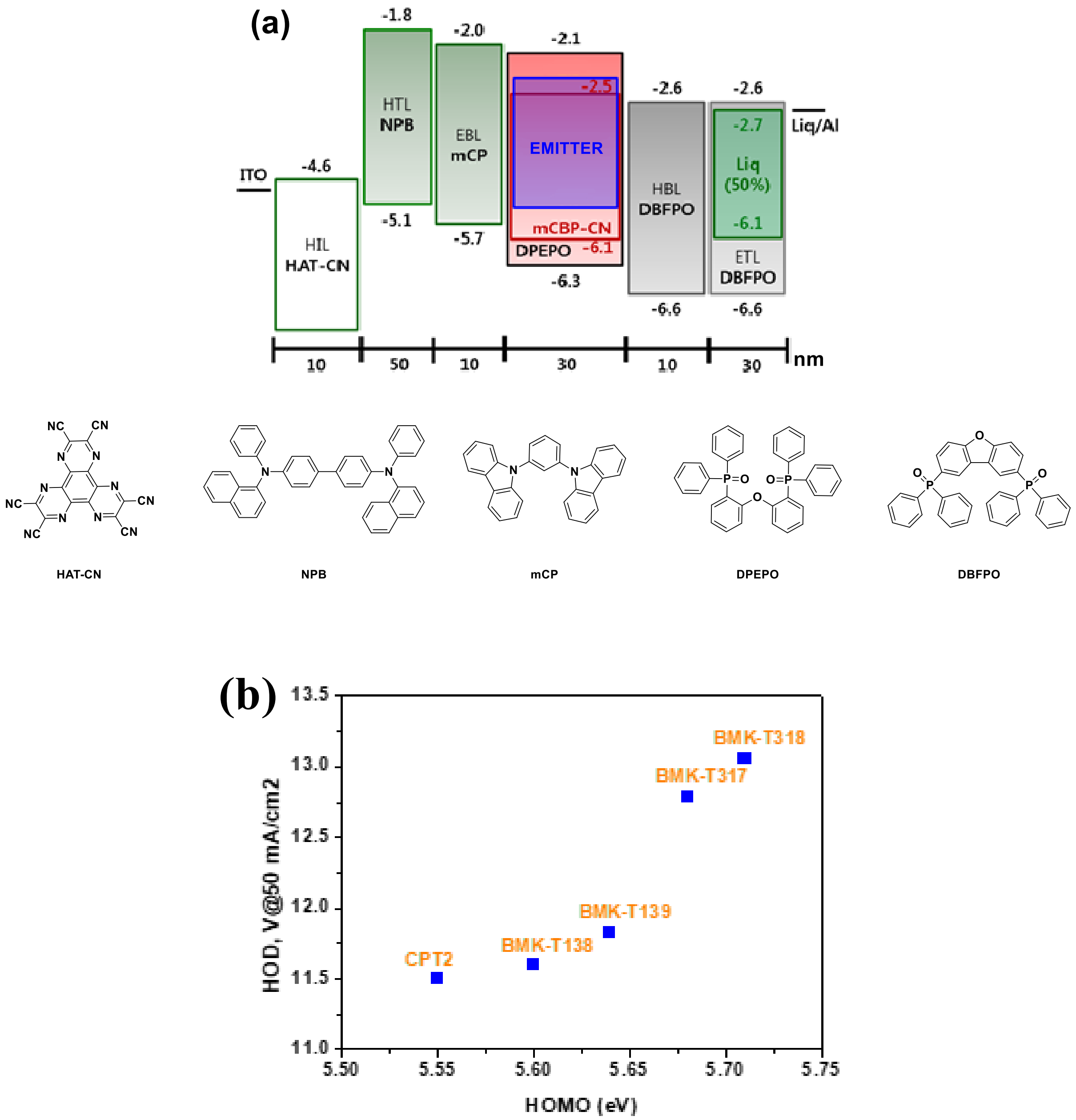

Figure S5. (a) Chemical structures and energy diagram of TADF devices (b) hole current versus HOMO energy.

Table S1. Additional theoretical computations data of the TADF emitters

TADF Emitter	SOC[a]	Reorg E[b]
**BMK-T138**	0.318	0.056
**BMK-T139**	0.312	0.074
**BMK-T317**	0.357	0.123
**BMK-T318**	0.316	0.118

a. Spin-orbit couplings., b. Reorganization energy

The SOC and Reorganization energy values are given below. And this was added in the revised supporting information (**Table S1**) as shown below. The $_{S}$ of the TADF emitters was affected with the help of the triazine backbone acceptor because they can restrict the molecular motion by steric hindrance. This can be easily predicted by the excited state geometry of the four emitters in S1 and T1 states. The improved SOC matrix element value of **BMK-T317** indicates that this emitter has dominant local excited (LE) state. Although the other three TADF emitters showed small overlap values because of the dominant charge transfer (CT) state, **BMK-T317** showed large overlap value in the excited state because of the dominant LE state.

Table S2. Measured HOMO-LUMO energy levels of the TADF emitters

TADF Emitter	HOMO (eV)	LUMO (eV)
**BMK-T138**	-5.60	-2.78
**BMK-T139**	-5.64	-2.72
**BMK-T317**	-5.68	-2.78
**BMK-T318**	-5.71	-2.82

The energy levels were measured by using cyclic voltammetry (CV). Each material was dissolved in anhydrous dichloromethane with 0.1 M tetrabutylammonium hexafluorophosphate as an electrolyte to measure the oxidation from which the HOMO energy level was estimated. A glassy carbon was used as the working electrode, a platinum wire was used as a counter electrode, and a saturated Ag/AgCl was used as a reference electrode Ferrocene was used as the standard reference. All solutions were purged with nitrogen for 10 minutes before each experiment. The LUMO levels of each materials were estimated from the difference between the HOMO energy level and the optical band gap which were estimated from the absorption edge of the UV-Vis spectra as shown in the revised supporting information (Table S2).
